# Peripubertal Nutritional Prevention of Cancer-Associated Gene Expression and Phenotypes

**DOI:** 10.3390/cancers15030674

**Published:** 2023-01-21

**Authors:** Andrew Brane, Itika Arora, Trygve O. Tollefsbol

**Affiliations:** 1 Department of Biology, University of Alabama at Birmingham, Birmingham, AL 35233, USA; 2 Department of Anesthesiology and Perioperative Medicine, University of Alabama at Birmingham, Birmingham, AL 35294, USA; 3 O’Neal Comprehensive Cancer Center, University of Alabama at Birmingham, Birmingham, AL 35294, USA; 4 Integrative Center for Aging Research, University of Alabama at Birmingham, Birmingham, AL 35294, USA; 5 Nutrition Obesity Research Center, University of Alabama at Birmingham, Birmingham, AL 35294, USA; 6 Comprehensive Diabetes Center, University of Alabama at Birmingham, Birmingham, AL 35294, USA; 7 University Wide Microbiome Center, University of Alabama Birmingham, Birmingham, AL 35294, USA

**Keywords:** breast cancer, puberty, critical periods, cancer prevention, nutrition, gene expression, DNA methylation

## Abstract

**Simple Summary:**

Certain life stages, known as critical periods, during growth and development are thought to be important for later-life breast cancer initiation and progression. Nutritional factors, especially those found in plant-based diets, are believed to be key to the impact of these critical periods on cancer. However, there is currently little known with respect to how nutrition during critical periods can affect breast cancer. In this study we evaluated nutritional intervention during the critical period of puberty and whether it could have a significant effect on tumor phenotype, as well as underlying gene expression, protein expression and DNA methylation patterns. We found that sulforaphane-containing broccoli sprout extracts administered during the peripubertal period in mice were able to reduce tumor size and incidence while delaying latency. We also found gross changes to gene expression patterns, including many cancer-associated genes, as well as potentially important changes to methylation profiles in our treatment groups.

**Abstract:**

Breast cancer (BC) is a nearly ubiquitous malignancy that effects the lives of millions worldwide. Recently, nutritional prevention of BC has received increased attention due to its efficacy and ease of application. Chief among chemopreventive compounds are plant-based substances known as dietary phytochemicals. Sulforaphane (SFN), an epigenetically active phytochemical found in cruciferous vegetables, has shown promise in BC prevention. In addition, observational studies suggest that the life stage of phytochemical consumption may influence its anticancer properties. These life stages, called critical periods (CPs), are associated with rapid development and increased susceptibility to cellular damage. Puberty, a CP in which female breast tissue undergoes proliferation and differentiation, is of particular interest for later-life BC development. However, little is known about the importance of nutritional chemoprevention to CPs. We sought to address this by utilizing two estrogen receptor-negative [ER(-)] transgenic mouse models fed SFN-containing broccoli sprout extract during the critical period of puberty. We found that this treatment resulted in a significant decrease in tumor incidence and weight, as well as an increase in tumor latency. Further, we found significant alterations in the long-term expression of cancer-associated genes, including *p21*, *p53*, and *BRCA2*. Additionally, our transcriptomic analyses identified expressional changes in many cancer-associated genes, and bisulfite sequencing revealed that the antiproliferation-associated gene *Erich4* was both hypomethylated and overexpressed in our experimental group. Our study indicates that dietary interventions during the CP of puberty may be important for later-life ER(-) BC prevention and highlights potential important genetic and epigenetic targets for treatment and study of the more deadly variants of BC.

## 1. Introduction

Breast cancer (BC) is a widespread malignancy and major source of financial, social, and medical hardship in the United States. BC is expected to account for ~15% of all cancer cases in women, and trails behind only lung cancer in terms of female cancer mortality [1,2]. Despite this, BC generally has favorable survival outcomes when compared to other cancer types. While many increases in survival are attributed to advances in early detection and treatments, the large remaining disparity between survival outlook and mortality is due to differences in disease severity among molecular subtypes of BC [3]. These subtypes are commonly categorized by the receptors present on the cell surface and include Luminal A, Luminal B, HER2-enriched, and triple negative (TNBC). As many of the most efficacious treatments are targeted to these receptors, the receptor-poor HER2 and receptor-negative TNBC are the most deadly and difficult to treat [3,4].

Interest in a cure for BC remains high, with over 500 clinical trials sponsored by the NIH today [5]. However, true cures for the deadliest forms of BC remain elusive, and successful treatment of any case of BC can result in a wide array of deleterious side effects, including loss of bone density, neuropathy, and cognitive decline [6,7,8]. Further, treatment can also result in financial hardships due to both the cost of treatment and loss of the ability to work post-therapy [9]. For these reasons, BC control through prevention is an appealing target for study that has the potential to minimize human suffering that arises from the disease.

Historically, cancer prevention has primarily been accomplished through avoidance of risk factors associated with specific cancer types, such as tobacco use for lung and oral cancers or alcohol consumption for stomach, breast, and prostate cancers [10,11]. However, cancer prevention can also be achieved though chemoprotective compounds administered before the onset of the disease. The two most common drugs used for BC prevention are Tamoxifen and Raloxifene, and both have been shown to reduce BC risk by around 40% [12]. However, these drugs can also lead to severe side effects, including induction of menopausal symptoms, blood clots, and, in the case of Tamoxifen, increased risk of uterine cancer. As a result, these drugs are often only prescribed to high-risk individuals for the purpose of cancer prevention.

Because of this, preventive interventions with wider scopes of use are in high demand. In particular, nutritional prevention of BC is a growing field that has potential for widespread use that is relatively inexpensive and efficacious with few to no side effects [13]. A group of chemopreventive compounds of high interest are those found within a plant-based diet and are collectively termed dietary phytochemicals. Sulforaphane (SFN), a dietary phytochemical found within cruciferous vegetables, has been shown to be efficacious in the prevention of BC within multiple mouse models [14]. These results are backed by clinical studies that inventoried the nutritional habits of BC patients and women in the wider population [15].

A major question that remains regarding nutritional prevention of BC concerns the timing of nutritional interventions. Specifically, it has been suggested that there are certain windows of susceptibility in which breast tissue is both more vulnerable to damage and responsive to preventive measures [16,17,18]. For breast tissue, growth and development primarily occurs during the prenatal period, puberty, and time of first pregnancy [19,20]. Environmental exposures during these windows, termed critical periods (CPs) in the context of cancer development, include prenatal exposure to the miscarriage prevention drug diethylstilbestrol, as well as peripubertal/peripregnancy exposure to the pesticide DDT, and have been linked to increased risk for BC development [21,22,23]. The CP of puberty has drawn particular interest due to its close association with rapid breast development, as well as fluctuations in hormone levels, which are important for the development of many types of BC [24].

Initial studies most commonly linked early onset of puberty to increased BC risk, reasoning that an increased duration of hormonal exposure resulted in increased BC risk [18,24,25]. It has also been reported that specific foods consumed during puberty may have an effect on later-life BC development [26]. Specifically, there are some indications that diets high in fats during puberty may increase BC risk later in life [27]. Few studies, however, have explored how peripubertal diet can decrease risk for BC, and fewer still have evaluated this in a basic science setting.

**Figure 1:**
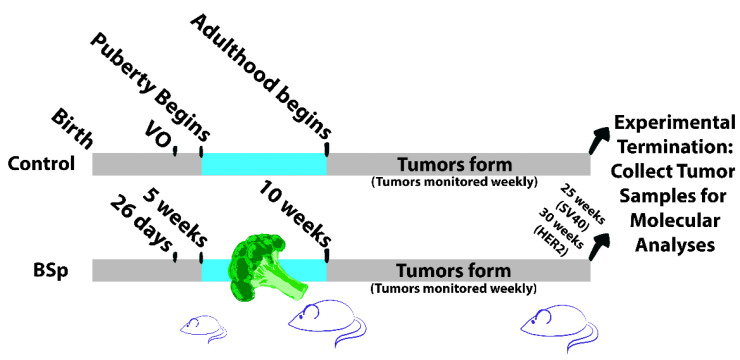
Overview of experimental design. Experiments were conducted in both SV40 and HER2/neu transgenic mouse lines. Puberty in mice begins about 10 days following vaginal opening (VO). Mice in the experimental (BSp) group received chow infused with SFN-containing BSp for a total of 5 weeks, beginning on the first day of the 5th week and ending on the last day of the 9th week. Mice were monitored for tumor formation beginning at 10 weeks of age. Experiments were terminated and tumor samples were collected for downstream analysis when tumor size reached 1 cm^3^. For all groups *n* = 24.

In this study, we utilized two transgenic mouse models for estrogen receptor-negative BC to elucidate the effects of SFN-containing broccoli sprout extract (BSp) administered during the critical period of puberty. Our aim was to determine whether or not BSp given during only the peripubertal period could have a long-term effect on BC tumor morphology, gene expression, and DNA methylation. We hypothesized that peripubertal BSp treatment would result in a decrease in tumor size and number and an increased latency period, as well as having long-term effects on both gene expression and global methylation patterns when compared to a standard diet during this time. To test this, we fed our mouse models SFN-containing BSp over a 5-week peripubertal period and measured tumor characteristics throughout life, as well as molecular changes that occurred at experimental termination. An overview of our experimental design is outlined in Figure 1 below.

## 2. Materials and Methods

### 2.1. Animal Housing and Husbandry

#### 2.1.1. Animal Housing and Experimental Design

All mice were housed in the UAB Campbell Hall animal facility. Mice were bred at around 8–10 weeks of age and weaned at 21 days old, with genotyping performed at the time of weaning. Mice were fed and given water ad libitum. At five weeks of age, experimental group mice were given the BSp diet until 10 weeks of age. During this time, control mice were continuously fed with NIH-31 variety mouse chow. For both experimental and control groups, *n* = 24. This treatment window lasted a total of 5 weeks. Beginning at 10 weeks, both groups were fed NIH-31 variety. Beginning after weaning, puberty was monitored daily and confirmed in all females through observation of vaginal opening (VO). VO is a readily observable characteristic that occurs 7–10 days prior to the first ovulation in mice [28,29]. While mice typically reach sexual maturity between 6–8 weeks of age, additional behavioral and developmental changes continue to occur until around 10 weeks of age [30]. Throughout their lives, tumor size and incidence were measured in each individual on a weekly basis. For in vivo experiments *n* = 24.

#### 2.1.2. Transgenic Mouse Lines

The C3(1)-SV40 Tag (FVB-Tg(C3-1-TAg)cJeg/JegJ) (SV40) mouse line typically develops tumors resembling Ductal Carcinoma in situ (DCIS) within the mammary epithelium at approximately 15 weeks of age, with 100% of female mice developing tumors by around 6 months of age [31]. The FVB/N-Tg(MMTVneu)202Mu (Her2/neu) Her2/neu mouse line develops ER(-) mammary tumors beginning at 20 weeks of age with a median age of 30 weeks [32]. Both were available from the Jackson Laboratories as breeder pairs from 4 weeks of age.

#### 2.1.3. Animal Diet

Chow infused with 26% broccoli sprouts (BSp) is commercially available from TestDiet and was produced by infusing control chow with BSp. The BSp was obtained through Natural Sprout Company (Springfield, MO) and was infused into pellets by TestDiet (Branchburg, NJ). This amount was equivalent to the consumption of 266 g (~4 cups) of BSp per day in humans, and this amount has already been shown to be both realistic and efficacious [33]. Total SFN content for BSp food at the concentration used (26%) is between 5.13 and 6.60 μM per gram of BSp [34]. Full information on the contents of experimental food is available in Supplementary Data S1. Control chow is the AIN-93G variety, and both BSp and control chow are administered ad libitum, with no detectable difference in the total volume of chow consumed. Diets were confirmed to have no effect on oncogenic driver expression.

#### 2.1.4. Tissue Collection

Upon experiment termination, mice were sacrificed using CO_2_ according to ARP protocols. Breast tumor samples for experimental protocols were collected subdermally, flash frozen, and stored at −80 °C. Tumor weight was recorded at the time of termination. For potential future experiments, additional blood samples were collected through an intracardiac puncture along with normal breast tissue that was collected subdermally. Organ tissues were collected from the thoracic cavity. All samples were stored at −80 °C.

### 2.2. Nucleic Acid Extraction and Analysis

#### 2.2.1. DNA and RNA Extraction

All nucleic acid extractions were performed on frozen breast tumor samples from control and BSp-fed groups. Total RNA for qPCR was extracted utilizing a Qiagen RNeasy kit per the manufacturer’s instructions. Total RNA for sequencing was extracted using TRIzol reagent based on the manufacturer’s protocols. Genomic DNA was extracted using the Qiagen DNEasy kit according to the manufacturer’s instructions. All nucleic acids were assessed for purity and concentration using a Nanodrop spectrophotometer.

#### 2.2.2. qPCR

cDNA was synthesized per the manufacturer’s instructions from 250 ng of RNA using iScript Reverse Transcription Supermix for RT–qPCR (BIORAD). Using the cDNA generated from this protocol, primers obtained from Integrated DNA Technologies, Inc. (Coralville, IA, USA), and SsoAdvanced Universal SYBR^®^ Green Supermix (BIORAD, Hercules, CA, USA), quantitative real-time PCR was performed. These reactions were performed in triplicate using the CFX Connect Real-Time PCR Detection System (BIORAD). Thermal cycling began at 94 °C and was followed by 35 cycles of PCR (94 °C for 15 s, 60 °C for 30 s, 72 °C for 30 s). *GAPDH* served as an endogenous control, and a vehicle control was used for calibration. Relative changes in gene expression were calculated through the 2-∆∆CQ method, where ∆∆CQ = [∆CQ(treatment group) − ∆CQ(control group)] and ∆CQ = [CQ(gene of interest)-CQ(GAPDH)] [35]. Relative expression levels of these genes were compared between treatment and control groups. For all qPCR experiments, *n* = 10. A full list of primers can be found in Supplementary Table S1.

#### 2.2.3. Western Blotting

Total protein from around 50 mg of flash-frozen mammary tumors was extracted with T-PER Tissue Protein Extraction Reagent (Thermo Fisher Scientific, St. Louis, MO, USA), according to the manufacturer’s protocol. Protein concentrations were ascertained utilizing a Bradford Assay, and denatured samples were subjected to electrophoresis on 4–15% NuPAGE Tris-HCl precast gels (Invitrogen, Waltham, MA, USA). Proteins were transferred onto nitrocellulose membranes and subsequently probed with antibodies to p21, p53, and BRCA2. Actβ was used as the loading control for each membrane. Antibody details can be found in Supplementary Table S2. Protein bands were visualized using Clarity Max™ Western ECL Blotting Substrates (Bio-Rad, Hercules, CA, USA) on a ChemiDoc™ XRS + System (Bio-Rad). Protein expression was quantified using ImageJ. For Western Blot experiments *n* = 6.

#### 2.2.4. RNA Sequencing

RNA sequencing was performed in a manner similar to previous work in our laboratory on tumor samples taken from control and BSp-fed groups [36]. RNA-seq was performed on extracted RNA by the UAB Heflin Genomics Core utilizing an Illumina NextSeq500 (Illumina, San Diego, CA, USA). Samples were assessed for quality using FastQC (v0.11.4) and aligned to the mouse reference genome GRCm38/mm10 using the default parameter settings of Kallisto. Further BAM file processing was performed with Kallisto, and transcription-level abundance estimates were generated for each sample file [37]. Following this, these estimates were input into the tximport package in R, allowing for gene-level expressional analysis [36,38]. Identification of differentially expressed genes (DEGs) was conducted with the Limma package in R, wherein the significant threshold for DEGs was set to |log2(fold-change)| >  2 and false discovery rate (FDR)  ≤  0.01. To identify enriched pathways, we utilized the web-based gene ontology analysis tool WebGestalt with our enriched gene list [39]. For RNAseq analyses, *n* = 7.

#### 2.2.5. Reduced Representation Bisulfite Sequencing (RRBS)

RRBS was performed similarly to previous work in our laboratory on breast tumor samples taken from both control and BSp-fed groups, and pair-end libraries were generated and sequenced by the UAB Heflin genomics core using an Illumina NextSeq500 [36]. Samples were assessed for quality using FastQC and trimmed using trim_galore based on the NuGEN Ovation RRBS system. These reads were aligned to the aforementioned mouse genome using Bismark alignment with default parameter settings. Utilizing the bismark_methylation extractor, CpG site call files were generated.

Analysis of differentially methylated regions and genes (DMRs and DMGs) was conducted with the methylKit package in R (v 3.6.1) utilizing the call files generated from the Bismark_methylation extractor [36,40]. DMRs and DMGs were identified based on a false discovery rate of ≤0.05, and methylation profiles between the control and BSp-treated group were generated through hierarchical clustering with the hclust package in R.

To build on our understanding of the association between methylation and gene expression, identified DMRs were analyzed for correlation with DEGs. DMR-DEG pairs that were significantly correlated (*p* < 0.05) were identified. For RRBS, *n* = 7.

### 2.3. Statistical Analyses

For all experiments, the statistical significance of expression differences, as well as tumor latency and size between experimental and control samples were determined using a Student’s T-test performed in Microsoft Excel. For tumor incidence, additional tests for significance were performed using a Chi-Squared test in SPSS statistical software (IBM) [34]. For all tests, a cutoff of *p* < 0.05 was considered statistically significant, with *p* < 0.05 being indicated by *, *p* < 0.01 being indicated by **, and *p* < 0.001 being indicated by ***. A minimum sample size of 11 was calculated using the 2-Sample, 1-Sided online power calculator found at powerandsamplesize.com [41]. For this calculation, a power of 0.8 and a significance of *p* = 0.05 were used.

## 3. Results

### 3.1. BSp Administration during the Peripubertal Period Resulted in a Decrease in Mammary Tumor Formation in Both SV40 and HER2/neu Mice

**Figure 2:**
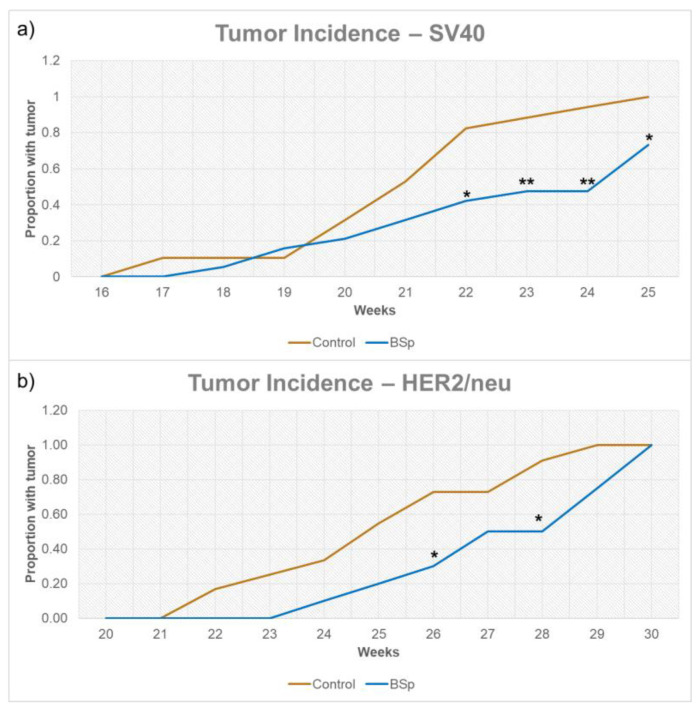
Tumor incidence for SV40 (**a**) and HER2/neu (**b**) mice. Tumor formation began around 16 weeks for SV40 mice and 21 weeks for HER2/neu mice. For both controls and experimental mice in both SV40 and HER2 experiments *n* = 24 mice. * = *p* < 0.05 and ** = *p* < 0.01.

**Figure 3:**
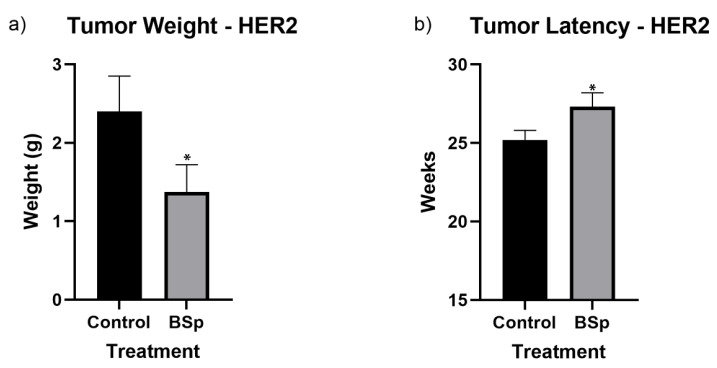
Tumor weight (**a**) and latency (**b**) of HER2/neu mice. BSp treatment significantly decreased mean tumor weight by 0.9 g and significantly increased mean tumor latency. For each group *n* = 24 mice. * = *p* < 0.05.

As depicted in Figure 2, overall tumor incidence for both SV40 and HER2/neu mice was significantly reduced for mice treated with BSp-infused chow. In SV40 mice, tumor formation began around 16 to 17 weeks in controls, with a more noticeable separation between control and treatments groups occurring after 21 weeks. In HER2/neu mice, tumor formation began around 21–22 weeks in controls, with a gap in incidence forming around 26 weeks. However, as HER2/neu mice reached 100% incidence, this separation closed. For HER2/neu mice, peripubertal BSp treatment also resulted in a significant decrease in tumor weight of approximately 0.9 g (Figure 3a) and a significant increase in tumor latency (Figure 3b). In SV40 mice, tumor latency followed a similar trend, but was not at significant levels (Supplementary Figure S1) and tumor weight was insignificant. In addition, BSp treatment had no significant effect on overall body weight (Supplementary Figure S2) or the timing of VO (Supplementary Figure S3). For both breeds of mice, the approximate tumor size was larger in control mice throughout life (Supplementary Figures S4 and S5).

### 3.2. BSp Administration during the Peripubertal Period Resulted in an Increase in Gene Expression of Key Cancer-Associated Genes in HER2/neu Mice

**Figure 4:**
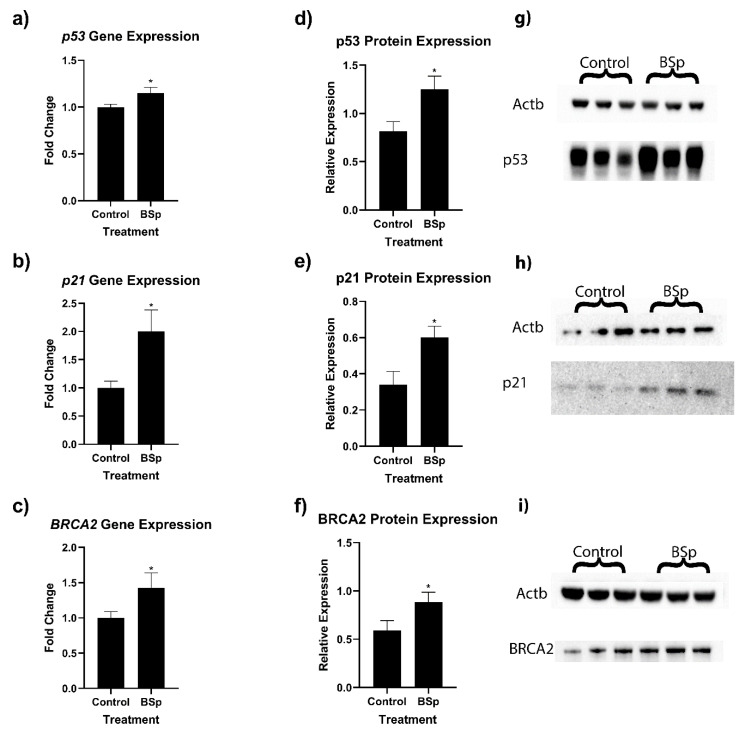
Expression of key tumor suppressor genes in HER2/neu mouse breast tumors. We observed significant increases in relative gene expression in *p53 (***a**), *p21* (**b**), and *BRCA2* (**c**). We also observed significant increases in protein expression for p53 (**d**,**g**), p21 (**e**,**h**), and *BRCA2* (**f**,**i**). For RT-qPCR, *n* = 10, and for Western blots, *n* = 6. * = *p* < 0.05. The uncropped blots are shown in File S1.

In order to ascertain how molecular mechanisms may be affecting our observed changes in tumor incidence, we performed RT-qPCR and Western blot analyses on key cancer-associated genes (Figure 4). Because the HER2/neu mice had a more robust response to BSp treatment, further molecular analyses were conducted on HER2/neu tumor samples. We found that in HER2/neu tumor samples, *p21* (a), *p53* (b), and *BRCA2* (c), gene expressions were significantly upregulated. We also found significant increases in the protein expression of both p53 (d and g), p21 (e and h), and BRCA2 (f and i). We also evaluated expressional changes in *BRCA1* and *tert*, but there was no significant difference in expression levels.

### 3.3. BSp Administration during the Peripubertal Period in HER2/neu Mice Resulted in Gross Changes to Gene Expression Profiles, with Effects on Many Key Cancer-Associated Genes and Pathways

To achieve a more wholistic view of gene expression changes, as well as to identify potentially important candidate genes, we performed RNA-seq analyses. We found significant expressional changes in 174 genes in the BSp-treated group when compared to the control. Overall, there were 92 genes downregulated and 82 genes upregulated in the BSp-treated group, and the top 20 downregulated and upregulated genes ranked by gene-expression fold change are displayed in Table 1 and Table 2, respectively. Several candidate genes were selected for PCR verification, including candidate oncogenes *Chrdl2*, *Pcsk1*, and *Slc51b* from the downregulated gene list and candidate tumor suppressors *Lman1I*, *Clec4e*, and *Parp6* from the upregulated gene list (Supplementary Figure S6). A full list of genes along with their expressional changes is available in Supplementary Data S2.

**Figure 5:**
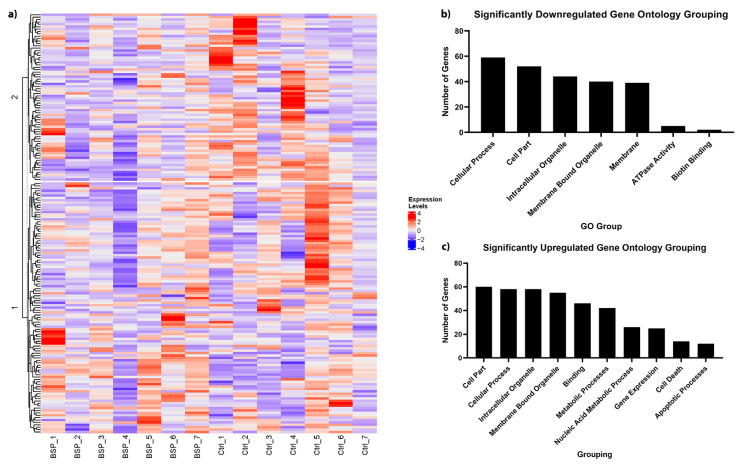
RNA sequencing heatmap (**a**), downregulated gene ontology (**b**), and upregulated gene ontology (**c**) analyses for breast tumor samples of peripubertal BSp-treated HER2/neu mice. For RNA-sequencing (**a**), *n* = 7 and genes were deemed significant if their adjusted *p*-value was *p* < 0.05. RNA sequencing results informed GO analyses in (**b**,**c**). For GO analyses, a cutoff of adjusted *p* < 0.05 was used to determine significant enrichment. The histograms shown here represent the GO groupings with the top 5 highest gene counts, as well as other groupings with physiological relevance to cancer biology.

To understand the biological processes that were affected by gene expression changes, we utilized our RNA sequencing results for gene ontology analysis. For both downregulated and upregulated genes, we identified significantly affected pathways (adj *p* < 0.05) in the categories of biological processes, molecular function, and cellular components. A summary of the five largest groupings, as well as those with known effects on cancer biology are outlined in Figure 5. These groupings are not mutually exclusive, and some genes were excluded due to a lack of information on their known biological function. A full list of genes within their respective GO groupings can be found in Supplementary Data S3 and S4 for downregulated and upregulated genes, respectively. In addition, full figures generated by the Webgestalt program are available as Supplementary Figures S7 and S8.

### 3.4. BSp Administration during the Peripubertal Period in HER2/neu Mice Had a Lasting Effect on Genome-Wide Methylation, and was Associated with Increases in Expression to the Anti-Proliferation Linked Erich4 Gene

**Figure 6:**
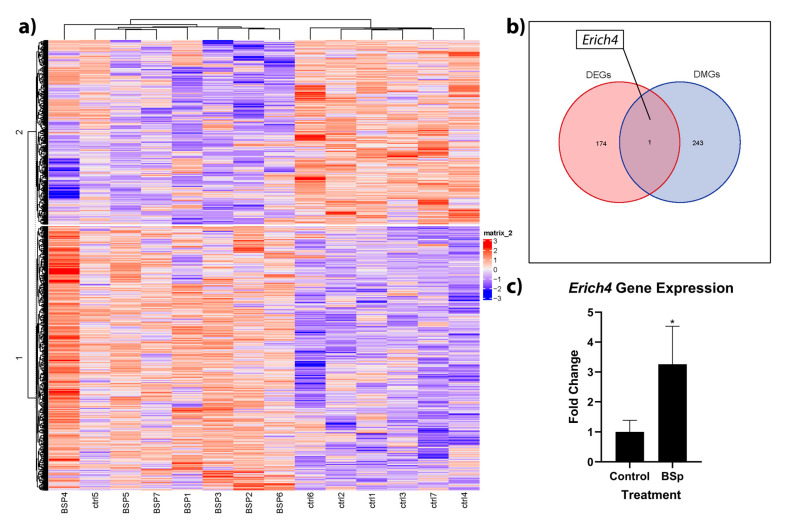
Heatmap of differentially methylated genes within breast tumor samples of BSp-treated mice vs. controls (**a**), as well as subsequent Venn diagram (**b**) of overlap between differentially expressed (red) and differentially methylated (blue) genes. *Erich4* was hypomethylated and overexpressed, and these results were verified with RT-qPCR (**c**). For RRBS and RNAseq, *n* = 7. For RT-qPCR, *n* = 10. * = *p* < 0.05.

To build on our understanding of how epigenetic effects may be playing a role in expressional and phenotypic changes, we followed our RNA sequencing work with whole genome RRBS analysis. Overall, we found CpG methylation changes in 243 genes, with increases in methylation levels for 113 genes and decreases in methylation for 130 genes in the BSp group relative to control (Figure 6a). To ascertain how these methylation changes may affect expression, we integrated our RNA sequencing and RRBS analyses. We found that out of the 243 differentially methylated genes and 174 differentially expressed genes, the gene *Erich4* was differentially methylated and expressed (Figure 6b). In the BSp-treated group, *Erich4* was both hypomethylated and overexpressed, and these high expression levels were verified with RT-qPCR (Figure 6c). A full list of differentially methylated genes is available in Supplementary Data S5.

## 4. Discussion

Despite continued advances in BC detection and therapies, BC mortality remains a leading cause of death for women worldwide. Preventive interventions have the potential to greatly reduce disease burden, thereby saving lives while simultaneously minimizing the economic strain associated with conventional therapies. In particular, nutrition-based prevention is relatively inexpensive, easy to implement, and has nearly no detectable negative side effects. Based on clinical observations of BC patients, there is evidence that nutritional prevention may be important at key life stages such as puberty [26]. Because the peripubertal period is a time in which nutritional compliance is feasible through school and parental supervision, understanding the relevance of this time period for later-life BC prevention may be vital for cancer control planning. Our work is among the first to study the effects of a known chemopreventive administered during puberty in a basic science setting. Our results indicate that SFN-containing BSp administered during puberty is sufficient to reduce tumor burden, which includes a significant decrease in size, a decrease in incidence, and an increase in latency, as well as having a profound effect on long-term gene expression. 

Earlier work from our lab indicates that BSp treatment has no significant effect on tumor incidence or latency when it begins to be administered at adulthood (defined in that study as beyond 8 weeks of age) and continues until termination [42]. This contrasts the effects witnessed in both SV40 and HER2/neu mice when BSp treatment is administered during the peripubertal period (beginning at 5 weeks of age and ending at 10 weeks of age). When compared to previous work in which BSp was administered throughout life, our data, as expected, showed more modest effects on tumor size, latency, and incidence [42]. However, our treatment window lasted only 5 weeks (vs. 29 weeks in the prior lifelong study), and our significant results indicate that BSp intervention during this period alone can result in significant decreases to both tumor burden and incidence. While the idea that pubertal diet can have an effect on later life BC development has been documented in clinical cohorts, a majority of these studies only examined high-fat diets as a mechanism of increased risk [26,27,43,44]. In addition to previous work from our laboratory, clinical observations have indicated that diets rich in cruciferous vegetables and, by extension, SFN have a chemoprotective effect, resulting in decreased chances of developing or dying from BC [34,42,45]. However, this study is among the first to show that intervention during critical periods such as puberty may be important for the prevention or delay of later-life BC development.

Along with observing these phenotypic changes, we also generated a unique expression profile for our peripubertal BSp treatment. To determine the potential for long-term gene expressional changes, we performed RT-qPCR on the BC-associated genes *p21*, *p53*, *BRCA1*, *BRCA2*, and *tert*. Our results indicated significant increases in expression for the tumor suppressor genes *p21*, *p53*, and *BRCA2* in HER2/neu mice. Protein validation found that there were significantly higher levels of p53, p21, and BRCA2 proteins at the time of experimental termination, indicating that these expressional changes were robust. Overall, these genes are closely tied to BC, and overexpression of each of these genes has importance for curbing BC development. Specifically, *p21* is not typically mutationally deactivated in the course of BC development, so upregulation of this gene may have preventive and therapeutic potential [46]. *p21* has also been shown to reduce BC burden and can be upregulated by the BC drug Valtrate [46,47,48]. *p53* is among the mostly widely studied tumor suppressor genes and its mutational loss is vital to approximately 35% of BC patients and 80% of TNBC cases [49]. Because of this, upregulation of a mutated *p53* is unlikely to achieve therapeutic success. However, from a prevention standpoint, upregulation of WT-*p53* in a precancerous cell could result in apoptotic destruction of the cell before full cancer cell transformation could occur. *BRCA2* is well known for its impact on BC, with its heritable mutation to either *BRCA1* or *BRCA2* being responsible for up to 10% of all BC cases in Western countries [50,51]. Much of the current body of research on *BRCA2* is associated with its loss, but lower expression levels are associated with decreased ability to repair DNA double-strand breaks, as well as an increased risk of developing BC [50,52]. In the context of our study, these three upregulated tumor suppressors have well documented anticancer effects, and it is likely that their increase in expression is responsible in part for the more favorable tumor characteristics we observed.

To build on our understanding of how peripubertal BSp treatment could affect gene expression patterns, we performed RNA sequencing. We identified 174 DEGs in the BSp-treated group, with a total of 82 upregulated and 92 downregulated genes. Within these groups, there are several genes that may be responsible for the phenotypic effects we observed, and several are known or are candidate tumor suppressors and oncogenes. 

Within our top 20 downregulated genes, our treatment had a significant long-term effect on the oncogenes *Chrdl2*, *Pcsk1*, and *Slc51b*. *Chrdl2* is an oncogene that has been associated with poor prognosis in colorectal cancer cells where it is known to be an inhibitor of apoptosis [53]. In BC, its overexpression is associated with increased capacity for bone metastases, and decreased expression results in decreased proliferative capacity of osteosarcoma cells [54]. Taken with our results, this suggests that BSp treatment during the peripubertal period could result in less severe BC outcomes, as well as fewer metastases. *Pcsk1* is over-expressed in breast and colorectal cancers, with poor prognoses being associated with this expression [55,56]. The current consensus is that *Pcsk1* is important for tumorigenesis, so downregulation of this gene may be important for the anticancer effects that we observed [55]. *Slc51b* is indicative of poor prognoses in hepatic cancer and its overexpression results in increased proliferation and invasion [57]. Downregulation of this gene with our BSp treatment may explain the differences we found in tumor size in our HER2/neu mouse lines.

Conversely, within the top 20 upregulated genes, our treatment had significant long-term effects on the tumor suppressors *Lman1I*, *Clec4e*, and *Parp6*. *Lman1l* is necessary for proper excretion of the angiogenesis and tumor growth inhibitor *A1AT*, and loss of *Lman1l* has been associated with both colorectal and prostate cancers [58,59]. While there is little current research on the importance of *Lman1l* in BC, its high expression combined with its known molecular function may explain the lower tumor weight we observed in BSp-treated HER2/neu mice. *Clec4e* is an important regulator of the immune response, and higher levels of its expression are correlated with increased immune cell infiltration in hepatocellular carcinoma [60]. As infiltration of CD4+ and γδ T cells is associated with better overall and disease-free survival in BC patients, our overexpression findings may explain the increase in latency in HER2/neu mice, as well as the decrease in incidence we observed in both mouse strains [61]. *Parp6* is a member of the PARP family, which are typically known as oncogenes [62]. However, *Parp6* has been shown to be a negative regulator of cell proliferation function via downregulation of Survivin in colorectal cancer and high *PARP6* expression has been correlated with better tumor cell differentiation [62,63]. If these effects extend to our BC model, this may explain a portion of the reduction in tumor size and incidence witnessed in our peripubertal treatment group.

For a more wholistic understanding of the pathways and cellular functions associated with DEGs we identified, we utilized WebGestalt, a web-based gene ontology toolkit [39]. For the 21 significantly downregulated gene pathways and 65 significantly upregulated pathways, there were several standout groups with respect to cancer biology. Within the downregulated genes we discovered that both ATPase activity pathways (five genes) and biotin binding pathways (two genes) were significantly enriched. High expression of vacuolar ATPases have been implicated in cancer cell survival, development of drug resistance, and metastasis [64]. Interestingly, *Wrn*, *Abcd4*, *Ythdc2*, and *Dync1h1* within this group are noted to have oncogenic function in colon, breast, gastric, and colorectal cancers, respectively [65,66,67,68]. Downregulation of these genes and this pathway may explain the differences we found between the control and peripubertal BSp-treatment groups. Biotin can act as an alternate energy source to sustain tumor cell proliferation and biotin buildup, and increased transporter expression often occurs in cancer cells, including BC cells [69,70]. Because of this, biotin-bound molecules can also more easily enter cancer cells. In our study, we observed downregulation of biotin-binding genes *HLCS* and *ACACA*. *HLCS* is a gene important for biotin transport and is predictive of lymph node metastases and poor prognosis in BC [69]. In glioblastoma xenograft models its overexpression is predictive of poor prognosis and depletion disrupts tumorigenicity [71]. *ACACA* is relatively more poorly understood, but downregulation in mouse models has been found to suppress prostate cancer progression and lower tumor volume [72]. Our treatment’s downregulation of these genes correlated with our anticancer in vivo results and were congruent with these studies, so it is possible that changes in expression of these genes are responsible, in part, for our observations.

Within our upregulated gene enrichment set, there was a large number of metabolic processes affected (42 genes) with a particular focus on gene sets that affect gene expression (25 genes) and nucleic acid metabolism (26 genes). Upregulation of these gene sets may give us some indication as to how peripubertal BSp treatment alone could have such a profound long-term effect on overall gene expression patterns. Possibly more significant, however, is our treatment’s effect on genes within the cell death grouping (14 genes) and its subset of apoptotic processes (12 genes). Several genes within this group, including *BLCAP*, *CCAR1*, and *TRIM39*, have expressions that are linked directly to apoptosis or cell cycle arrest [73,74,75]. Upregulation of these and other apoptosis-associated genes may explain the more favorable tumor phenotype we observed in the peripubertal BSp-treated group.

Finally, we sought to determine if peripubertal BSp treatment could have long-term effects on the methylome and what part, if any, these methylation changes had on our DEG profiles. Past work in our lab has implicated SFN within BSp as having a significant effect on DNMTs and global methylation patterns, both in vitro and in vivo [36,76]. We measured a total of 243 differentially methylated genes (DMGs), with hypermethylation in 113 genes and hypomethylation in 130 genes. Our results indicate that peripubertal BSp treatment does have an effect on methylation patterns. While there is in vitro evidence that SFN contained within BSp acts as a DNMT inhibitor; our results, as well as those of our previous work, indicate that in vivo effects of SFN are more complex, having both hypo- and hyper-methylating effects on the epigenome [36,76,77].

With 174 DEGs and 243 differentially methylated genes, we identified a key gene, *Erich4*, that was differentially methylated and expressed. Additionally, known as glutamate rich 4, *Erich4* is relatively poorly understood, but its low expression has been associated with renal cell carcinoma and its mutational loss has been recorded in basal cell carcinoma [78,79]. Interestingly, one of its few known interactions is with *PPP2R5A*, a gene implicated in negative control of cell growth [80]. If this interaction is important in stemming cancer cell proliferation, it is possible that the hypomethylation and overexpression of *Erich4* with BSp treatment contributed to the anticancer effects that we found in vivo.

This study provides an important first step in understanding how dietary phytochemicals, such as SFN-containing BSp, can provide long-term protective effects when administered during a CP. We observed significant reductions to tumor severity with peripubertal treatment, but future work remains on the impact of other CPs and their relative importance to overall BC prevention. We also observed changes in the expression of many genes, including known and potential cancer-associated genes, and further studies will be required to determine which of these genes are more important to tumor progression and morphology. Finally, our results indicate that our peripubertal treatment can have a significant impact on global DNA methylation patterns. Further study will be necessary in understanding the mechanistic basis of the expressional changes we observed.

## 5. Conclusions

Overall, we found long-term reductions in the cancer phenotype, as well as changes to gene expression and methylation profiles due to dietary intervention administered during puberty alone. While this is in accordance with previous work indicating that adolescent diet could have an effect on BC development, our study is among the first to indicate that a peripubertal intervention with dietary phytochemicals can have an effect on later-life BC development [26]. We also developed gene expression and methylation profiles for peripubertal BSp exposure. Within this profile we identified tumor suppressor genes, including *p21*, *p53*, and *BRCA2*, that were upregulated in HER2/neu BSp-treated mice. In addition to these genes, we identified a suite of upregulated and downregulated genes, many of which have known or potential tumor suppressor or oncogenic functions. Although we did not find a large number of both differentially methylated and expressed genes, global bisulfite sequencing did reveal a potential tumor suppressor, *Erich4*, that appears to be under epigenetic control. Taken together, our results indicate that nutritional prevention of later-life BC utilizing critical periods, such as puberty alone, is feasible. These results indicate that the administration of nutritional interventions for BC prevention may be key during critical periods such as puberty, and further clinical and laboratory studies of how substances, such as dietary phytochemicals, administered during these periods affect BC development and have the potential to inform decisions involving BC prevention and control.

## Figures and Tables

**Table 1:** Top 20 downregulated genes in BSp-treated breast tumor samples vs. control tumor samples. For both groups *n* = 7 mice and significance cutoff was an adjusted *p* value < 0.05.

Gene ID	Name	Log FC	Avg. Exp.	adj. *p* Value
*Pcdhb1*	Protocadherin Beta 1	−3.39609	−4.2328285	0.0008754
*Vmn2r29*	Vomeronasal 2, receptor 29	−2.91192	−4.2173375	0.04718332
*Gm21962*	Predicted gene, 21962	−2.86629	−3.8732011	0.02353836
*Gm28778*	Predicted gene 28778	−2.681	−4.5447846	0.0008754
*Mroh8*	Maestro Heat Like Repeat Family Member 8	−2.55521	−4.6539474	0.00107869
*Olfr798*	Olfactory receptor 798	−2.33029	−4.2038604	0.00553995
*Prss39*	Protease, serine 39	−2.30801	−4.0433775	0.04061123
*Olfr837*	Olfactory receptor 837	−2.27784	−4.3915511	0.00413192
*Olfr592*	Olfactory receptor 592	−2.21951	−4.1896308	0.00413192
*Olfr1181*	Olfactory receptor 1181	−2.21231	−4.669899	0.04061123
*Neurod6*	Neuronal Differentiation 6	−2.12643	−4.5476479	0.00802338
*Gm2897*	Predicted gene 2897	−2.11836	−4.4376761	0.04726518
*Slc51b*	Solute Carrier Family 51 Subunit Beta	−2.11418	−4.6957968	0.02815029
*Vmn1r210*	Vomeronasal 1 receptor 210	−2.11002	−4.4579116	0.02274392
*Olfr1228*	Olfactory receptor 1228	−2.06907	−4.8911034	0.00490394
*Pcsk1*	Proprotein convertase subtilisin/kexin type 1	−1.95999	−2.733677	0.00720816
*Srarp*	Steroid Receptor Associated And Regulated Protein	−1.9012	−4.7484372	0.04726518
*Hoxc10*	Homeobox C10	−1.89364	−4.2726565	0.01709571
*Chrdl2*	Chordin Like 2	−1.799	−4.4780566	0.04187643
*Slc6a21*	Solute carrier family 6 member 21	−1.77935	−5.1522347	0.02831963

**Table 2:** Top 20 upregulated genes in BSp-treated breast tumor samples vs. control tumor samples. For both groups *n* = 7 mice and significance cutoff was an adjusted *p* Value < 0.05.

Gene ID	Name	Log FC	Avg. Exp.	adj. *p* value
*Gm2237*	Predicted gene 2237	3.158839	−4.602845	0.0008754
*Lman1l*	Lectin, Mannose Binding 1 Like	2.951392	−4.1578837	0.00107869
*Clec4e*	C-Type Lectin Domain Family 4 Member E	2.886333	−3.9958048	0.01925175
*Kif19b*	Kinesin Family Member 19	2.583289	−4.5418484	0.0008754
*Armc12*	Armadillo Repeat Containing 12	2.483762	−4.2946274	0.00113373
*Tmem200c*	Transmembrane Protein 200C	2.018997	−4.6153797	0.02619552
*Olfr361*	Olfactory receptor 361	1.983424	−4.2906929	0.04288585
*Sncb*	Synuclein Beta	1.923413	−4.9915667	0.01746493
*Tmem59l*	Transmembrane Protein 59 Like	1.858611	−4.7316851	0.02557444
*Gm11168*	Predicted gene 11168	1.802597	−4.6737187	0.03114816
*Erich4*	Glutamate Rich 4	1.791582	−4.6379376	0.04726518
*Treml1*	Triggering Receptor Expressed On Myeloid Cells Like 1	1.710026	−4.8032762	0.01326325
*Olfr922*	Olfactory receptor 922	1.645667	−4.8070893	0.01625371
*Mmp13*	Matrix metallopeptidase 13	1.119203	1.333663	0.0463051
*Prmt6*	Protein Arginine Methyltransferase 6	1.092539	3.0074275	0.00815202
*Tspan4*	Tetraspanin 4	0.904403	5.6742821	0.04244361
*Sphk1*	Sphingosine Kinase 1	0.898623	4.7451435	0.01787144
*Parp6*	Poly(ADP-Ribose) Polymerase Family Member 6	0.806976	2.9441413	0.0113267
*Adam8*	ADAM metallopeptidase domain 8	0.779123	3.0242021	0.04726518
*Coq10b*	Coenzyme Q10B	0.622019	4.0602847	0.02931014

## Data Availability

Data are contained within the article or Supplementary Materials.

## Supplementary Materials

The following supporting information can be downloaded at: https://www.mdpi.com/article/10.3390/cancers15030674/s1. Data S1: Diet Composition; Data S2: Full DEG list; Data S3: GO Downregulated; Data S4: GO Upregulated; Data S5: Full DMR list; Table S1: Primer sequences for RT-Qpcr; Table S2: Antibodies for Western blots; Figure S1: Average tumor latency observed in SV40 mice; Figure S2: Mouse body weight for SV40 (a) and HER2/neu (b) mice; Figure S3: Average day of vaginal opening (VO) for SV40 (a) and HER2/neu (b) mice; Figure S4. Approximate tumor size for HER2/neu mice in both control (a) and BSp (b) fed mice; Figure S5. Approximate tumor size for SV40 mice in both control (a) and BSp (b) fed mice; Figure S6: PCR verification of tumor suppressors Lman1I (a), Clec4e (b), Parp6 (c), as well as oncogenes Chrdl2 (d), Pcsk1 (e), and Slc51b (f); Figure S7: Gene ontology analysis for downregulated genes identified by RNA sequencing; Figure S8: Gene ontology analysis for upregulated genes identified by RNA sequencing.

## References

[1] National Cancer Institute SEER Cancer Stat Facts: Female Breast Cancer. Bethesda, MD. https://seer.cancer.gov/statfacts/html/breast.html.

[2] American Cancer Society (2022). Cancer Facts & Figures 2022.

[3] Fragomeni S.M., Sciallis A., Jeruss J.S. (2018). Molecular Subtypes and Local-Regional Control of Breast Cancer. Surg. Oncol. Clin. N. Am..

[4] National Cancer Institute (2022). Hormone Therapy for Breast Cancer Fact Sheet. https://www.cancer.gov/types/breast/breast-hormone-therapy-fact-sheet.

[5] National Cancer Institute (2022). Treatment Clinical Trials for Breast Cancer. https://www.cancer.gov/about-cancer/treatment/clinical-trials/disease/breast-cancer/treatment.

[6] Collins B., Mackenzie J., Stewart A., Bielajew C., Verma S. (2009). Cognitive effects of chemotherapy in post-menopausal breast cancer patients 1 year after treatment. Psychooncology..

[7] Mayo Foundation for Medical Education and Research (2021). Chemotherapy for Breast Cancer. Mayo Clinic. https://www.mayoclinic.org/tests-procedures/chemotherapy-for-breast-cancer/about/pac-20384931.

[8] Greenlee H., DuPont-Reyes M.J., Balneaves L.G., Carlson L.E., Cohen M.R., Deng G., Johnson J.A., Mumber M., Seely D., Zick S.M. (2017). Clinical practice guidelines on the evidence-based use of integrative therapies during and after breast cancer treatment. CA Cancer J. Clin..

[9] Schmidt M.E., Scherer S., Wiskemann J., Steindorf K. (2019). Return to work after breast cancer: The role of treatment-related side effects and potential impact on quality of life. Eur. J. Cancer Care.

[10] Asthana S., Labani S., Kailash U., Sinha D.N., Mehrotra R. (2019). Association of Smokeless Tobacco Use and Oral Cancer: A Systematic Global Review and Meta-Analysis. Nicotine Tob. Res..

[11] de Menezes R.F., Bergmann A., Thuler L.C. (2013). Alcohol consumption and risk of cancer: A systematic literature review. Asian Pac. J. Cancer Prev..

[12] American Cancer Society (2021). Breast Cancer Prevention: Tamoxifen and Raloxifene. https://www.cancer.org/cancer/breast-cancer/risk-and-prevention/tamoxifen-and-raloxifene-for-breast-cancer-prevention.html.

[13] De Cicco P., Catani M.V., Gasperi V., Sibilano M., Quaglietta M., Savini I. (2019). Nutrition and Breast Cancer: A Literature Review on Prevention, Treatment and Recurrence. Nutrients.

[14] Nandini D.B., Rao R.S., Deepak B.S., Reddy P.B. (2020). Sulforaphane in broccoli: The green chemoprevention!! Role in cancer prevention and therapy. J. Oral Maxillofac. Pathol..

[15] Ullah M.F. (2015). Sulforaphane (SFN): An Isothiocyanate in a Cancer Chemoprevention Paradigm. Medicines.

[16] Krisanits B., Randise J.F., Burton C.E., Findlay V.J., Turner D.P. (2020). Pubertal mammary development as a "susceptibility window" for breast cancer disparity. Adv. Cancer Res..

[17] Cohn B.A., Cirillo P.M., Terry M.B. (2019). DDT and Breast Cancer: Prospective Study of Induction Time and Susceptibility Windows. J. Natl. Cancer Inst..

[18] Biro F.M., Deardorff J. (2013). Identifying opportunities for cancer prevention during preadolescence and adolescence: Puberty as a window of susceptibility. J. Adolesc. Health.

[19] Javed A., Lteif A. (2013). Development of the human breast. Semin. Plast. Surg..

[20] Johns Hopkins Medicine (2020). Normal Breast Development and Changes. https://www.hopkinsmedicine.org/health/conditions-and-diseases/normal-breast-development-and-changes.

[21] Cohn B.A., La Merrill M., Krigbaum N.Y., Yeh G., Park J.S., Zimmermann L., Cirillo P.M. (2015). DDT Exposure in Utero and Breast Cancer. J. Clin. Endocrinol. Metab..

[22] Terry M.B., Michels K.B., Brody J.G., Byrne C., Chen S., Jerry D.J., Malecki K.M.C., Martin M.B., Miller R.L., Breast Cancer and the Environment Research Program (BCERP) (2019). Environmental exposures during windows of susceptibility for breast cancer: A framework for prevention research. Breast Cancer Res..

[23] Hilakivi-Clarke L. (2014). Maternal exposure to diethylstilbestrol during pregnancy and increased breast cancer risk in daughters. Breast Cancer Res..

[24] Bodicoat D.H., Schoemaker M.J., Jones M.E., McFadden E., Griffin J., Ashworth A., Swerdlow A.J. (2014). Timing of pubertal stages and breast cancer risk: The Breakthrough Generations Study. Breast Cancer Res..

[25] Apter D. (1996). Hormonal events during female puberty in relation to breast cancer risk. Eur. J. Cancer Prev..

[26] Haraldsdottir A., Torfadottir J.E., Valdimarsdottir U.A., Adami H.O., Aspelund T., Tryggvadottir L., Thordardottir M., Birgisdottir B.E., Harris T.B., Launer L.J. (2018). Dietary habits in adolescence and midlife and risk of breast cancer in older women. PLoS ONE.

[27] Stoll B.A. (1998). Western diet, early puberty, and breast cancer risk. Breast Cancer Res. Treat..

[28] Caligioni C.S. (2009). Assessing reproductive status/stages in mice. Curr. Protoc. Neurosci..

[29] Nelson J.F., Felicio L.S., Randall P.K., Sims C., Finch C.E. (1982). A longitudinal study of estrous cyclicity in aging C57BL/6J mice: I. Cycle frequency, length and vaginal cytology. Biol. Reprod..

[30] Brust V., Schindler P.M., Lewejohann L. (2015). Lifetime development of behavioural phenotype in the house mouse (*Mus musculus*). Front. Zool..

[31] Maroulakou I.G., Anver M., Garrett L., Green J.E. (1994). Prostate and mammary adenocarcinoma in transgenic mice carrying a rat C3(1) simian virus 40 large tumor antigen fusion gene. Proc. Natl. Acad. Sci. USA.

[32] Guy C.T., Webster M.A., Schaller M., Parsons T.J., Cardiff R.D., Muller W.J. (1992). Expression of the neu protooncogene in the mammary epithelium of transgenic mice induces metastatic disease. Proc. Natl. Acad. Sci. USA.

[33] Li Y., Meeran S.M., Tollefsbol T.O. (2017). Combinatorial bioactive botanicals re-sensitize tamoxifen treatment in ER-negative breast cancer via epigenetic reactivation of ERα expression. Sci. Rep..

[34] Li S., Chen M., Wu H., Li Y., Tollefsbol T.O. (2020). Maternal Epigenetic Regulation Contributes to Prevention of Estrogen Receptor-negative Mammary Cancer with Broccoli Sprout Consumption. Cancer Prev. Res..

[35] Livak K.J., Schmittgen T.D. (2001). Analysis of relative gene expression data using real-time quantitative PCR and the 2(-Delta Delta C(T)) Method. Methods.

[36] Arora I., Li Y., Sharma M., Crowley M.R., Crossman D.K., Li S., Tollefsbol T.O. (2021). Systematic integrated analyses of methylomic and transcriptomic impacts of early combined botanicals on estrogen receptor-negative mammary cancer. Sci. Rep..

[37] Bray N.L., Pimentel H., Melsted P., Pachter L. (2016). Near-optimal probabilistic RNA-seq quantification. Nat. Biotechnol..

[38] Soneson C., Love M.I., Robinson M.D. (2015). Differential analyses for RNA-seq: Transcript-level estimates improve gene-level inferences. F1000Research.

[39] Liao Y., Wang J., Jaehnig E.J., Shi Z., Zhang B. (2019). WebGestalt 2019: Gene set analysis toolkit with revamped UIs and APIs. Nucleic Acids Res..

[40] Akalin A., Kormaksson M., Li S., Garrett-Bakelman F.E., Figueroa M.E., Melnick A., Mason C.E. (2012). methylKit: A comprehensive R package for the analysis of genome-wide DNA methylation profiles. Genome Biol..

[41] Hickey G.L., Grant S.W., Dunning J., Siepe M. (2018). Statistical primer: Sample size and power calculations-why, when and how?. Eur. J. Cardiothorac. Surg..

[42] Li Y., Buckhaults P., Li S., Tollefsbol T. (2018). Temporal Efficacy of a Sulforaphane-Based Broccoli Sprout Diet in Prevention of Breast Cancer through Modulation of Epigenetic Mechanisms. Cancer Prev. Res..

[43] Haslam S.Z., Schwartz R.C. (2011). Is there a link between a high-fat diet during puberty and breast cancer risk?. Women’s Health.

[44] Linos E., Willett W.C., Cho E., Frazier L. (2010). Adolescent diet in relation to breast cancer risk among premenopausal women. Cancer Epidemiol. Biomark. Prev..

[45] Farvid M.S., Holmes M.D., Chen W.Y., Rosner B.A., Tamimi R.M., Willett W.C., Eliassen A.H. (2020). Postdiagnostic Fruit and Vegetable Consumption and Breast Cancer Survival: Prospective Analyses in the Nurses’ Health Studies. Cancer Res..

[46] Diab S.G., Yu Y.Y., Hilsenbeck S.G., Allred D.C., Elledge R.M. (1997). WAF1/CIP1 protein expression in human breast tumors. Breast Cancer Res. Treat..

[47] Shamloo B., Usluer S. (2019). p21 in Cancer Research. Cancers.

[48] Li Y., Huang J., Zeng B., Yang D., Sun J., Yin X., Lu M., Qiu Z., Peng W., Xiang T. (2018). PSMD2 regulates breast cancer cell proliferation and cell cycle progression by modulating p21 and p27 proteasomal degradation. Cancer Lett..

[49] Duffy M.J., Synnott N.C., Crown J. (2018). Mutant p53 in breast cancer: Potential as a therapeutic target and biomarker. Breast Cancer Res. Treat..

[50] Mehrgou A., Akouchekian M. (2016). The importance of BRCA1 and BRCA2 genes mutations in breast cancer development. Med. J. Islam. Repub. Iran.

[51] Tereschenko I.V., Basham V.M., Ponder B.A., Pharoah P.D. (2002). BRCA1 and BRCA2 mutations in Russian familial breast cancer. Hum. Mutat..

[52] Bracci M., Ciarapica V., Zabaleta M.E., Tartaglione M.F., Pirozzi S., Giuliani L., Piva F., Valentino M., Ledda C., Rapisarda V. (2019). BRCA1 and BRCA2 Gene Expression: Diurnal Variability and Influence of Shift Work. Cancers.

[53] Sun J., Liu X., Gao H., Zhang L., Ji Q., Wang Z., Zhou L., Wang Y., Sui H., Fan Z. (2017). Overexpression of colorectal cancer oncogene CHRDL2 predicts a poor prognosis. Oncotarget.

[54] Chen H., Pan R., Li H., Zhang W., Ren C., Lu Q., Chen H., Zhang X., Nie Y. (2021). CHRDL2 promotes osteosarcoma cell proliferation and metastasis through the BMP-9/PI3K/AKT pathway. Cell Biol. Int..

[55] Demoures B., Siegfried G., Khatib A.-M. (2017). PCSK1 (Proprotein Convertase Subtilisin/Kexin Type 1) Atlas Genet Cytogenet Oncol Haematol. http://atlasgeneticsoncology.org/gene/41671/pcsk1.

[56] Chou C.L., Chen T.J., Lin C.Y., Lee S.W., Wang S.C., Chu S.S., Yang C.C. (2020). PCSK1 Overexpression in Rectal Cancer Correlates with Poor Response to Preoperative Chemoradiotherapy and Prognosis. OncoTargets Ther..

[57] Fang X., Liu Y., Xiao W., Zhao N., Zhu C., Yu D., Zhao Y. (2021). Prognostic SLC family genes promote cell proliferation, migration, and invasion in hepatocellular carcinoma. Acta Biochim. Biophys. Sin..

[58] Roeckel N., Woerner S.M., Kloor M., Yuan Y.P., Patsos G., Gromes R., Kopitz J., Gebert J. (2009). High frequency of LMAN1 abnormalities in colorectal tumors with microsatellite instability. Cancer Res..

[59] Lindberg J., Mills I.G., Klevebring D., Liu W., Neiman M., Xu J., Wikström P., Wiklund P., Wiklund F., Egevad L. (2013). The mitochondrial and autosomal mutation landscapes of prostate cancer. Eur. Urol..

[60] Zhang Y., Wei H., Fan L., Fang M., He X., Lu B., Pang Z. (2021). CLEC4s as Potential Therapeutic Targets in Hepatocellular Carcinoma Microenvironment. Front. Cell Dev. Biol..

[61] Zhang S.C., Hu Z.Q., Long J.H., Zhu G.M., Wang Y., Jia Y., Zhou J., Ouyang Y., Zeng Z. (2019). Clinical Implications of Tumor-Infiltrating Immune Cells in Breast Cancer. J. Cancer.

[62] Qi G., Kudo Y., Tang B., Liu T., Jin S., Liu J., Zuo X., Mi S., Shao W., Ma X. (2016). PARP6 acts as a tumor suppressor via downregulating Survivin expression in colorectal cancer. Oncotarget.

[63] Tuncel H., Tanaka S., Oka S., Nakai S., Fukutomi R., Okamoto M., Ota T., Kaneko H., Tatsuka M., Shimamoto F. (2012). PARP6, a mono(ADP-ribosyl) transferase and a negative regulator of cell proliferation, is involved in colorectal cancer development. Int. J. Oncol..

[64] Stransky L., Cotter K., Forgac M. (2016). The Function of V-ATPases in Cancer. Physiol. Rev..

[65] Liu B., Yi J., Yang X., Liu L., Lou X., Zhang Z., Qi H., Wang Z., Zou J., Zhu W.G. (2019). MDM2-mediated degradation of WRN promotes cellular senescence in a p53-independent manner. Oncogene.

[66] Hlaváč V., Václavíková R., Brynychová V., Koževnikovová R., Kopečková K., Vrána D., Gatěk J., Souček P. (2020). Role of Genetic Variation in ABC Transporters in Breast Cancer Prognosis and Therapy Response. Int. J. Mol. Sci..

[67] Yuan W., Chen S., Li B., Han X., Meng B., Zou Y., Chang S. (2022). The N6-methyladenosine reader protein YTHDC2 promotes gastric cancer progression via enhancing YAP mRNA translation. Transl. Oncol..

[68] Gong L.B., Wen T., Li Z., Xin X., Che X.F., Wang J., Liu Y.P., Qu X.J. (2019). DYNC1I1 Promotes the Proliferation and Migration of Gastric Cancer by Up-Regulating IL-6 Expression. Front. Oncol..

[69] Yoon J., Grinchuk O.V., Kannan S., Ang M.J.Y., Li Z., Tay E.X.Y., Lok K.Z., Lee B.W.L., Chuah Y.H., Chia K. (2021). A chemical biology approach reveals a dependency of glioblastoma on biotin distribution. Sci. Adv..

[70] Vadlapudi A.D., Vadlapatla R.K., Pal D., Mitra A.K. (2013). Biotin uptake by T47D breast cancer cells: Functional and molecular evidence of sodium-dependent multivitamin transporter (SMVT). Int. J. Pharm..

[71] Sukjoi W., Siritutsoontorn S., Chansongkrow P., Waiwitlikhit S., Polyak S.W., Warnnissorn M., Charoensawan V., Thuwajit C., Jitrapakdee S. (2020). Overexpression of Holocarboxylase Synthetase Predicts Lymph Node Metastasis and Unfavorable Prognosis in Breast Cancer. Anticancer Res..

[72] Zhang H., Liu S., Cai Z., Dong W., Ye J., Cai Z., Han Z., Liang Y., Zhuo Y., Luo Y. (2021). Down-regulation of ACACA suppresses the malignant progression of Prostate Cancer through inhibiting mitochondrial potential. J. Cancer.

[73] Zhao M., Zhang L., Qiu X., Zeng F., Chen W., An Y., Hu B., Wu X., Wu X. (2016). BLCAP arrests G₁/S checkpoint and induces apoptosis through downregulation of pRb1 in HeLa cells. Oncol. Rep..

[74] Johnson G.S., Rajendran P., Dashwood R.H. (2020). CCAR1 and CCAR2 as gene chameleons with antagonistic duality: Preclinical, human translational, and mechanistic basis. Cancer Sci..

[75] Zhang L., Mei Y., Fu N.Y., Guan L., Xie W., Liu H.H., Yu C.D., Yin Z., Yu V.C., You H. (2012). TRIM39 regulates cell cycle progression and DNA damage responses via stabilizing p21. Proc. Natl. Acad. Sci. USA.

[76] Royston K.J., Udayakumar N., Lewis K., Tollefsbol T.O. (2017). A Novel Combination of Withaferin A and Sulforaphane Inhibits Epigenetic Machinery, Cellular Viability and Induces Apoptosis of Breast Cancer Cells. Int. J. Mol. Sci..

[77] Zhou J.W., Wang M., Sun N.X., Qing Y., Yin T.F., Li C., Wu D. (2019). Sulforaphane-induced epigenetic regulation of Nrf2 expression by DNA methyltransferase in human Caco-2 cells. Oncol. Lett..

[78] Lenburg M.E., Liou L.S., Gerry N.P., Frampton G.M., Cohen H.T., Christman M.F. (2003). Previously unidentified changes in renal cell carcinoma gene expression identified by parametric analysis of microarray data. BMC Cancer.

[79] Sharpe H.J., Pau G., Dijkgraaf G.J., Basset-Seguin N., Modrusan Z., Januario T., Tsui V., Durham A.B., Dlugosz A.A., Haverty P.M. (2015). Genomic analysis of smoothened inhibitor resistance in basal cell carcinoma. Cancer Cell.

[80] Safran M., Rosen N., Twik M., BarShir R., Iny Stein T., Dahary D., Fishilevich S., Lancet D. (2022). The GeneCards Suite Chapter, Practical Guide to Life Science Databases. https://www.genecards.org/cgi-bin/carddisp.pl?gene=PPP2R5A.

