# Supplementary material for: Peripubertal Nutritional Prevention of Cancer-Associated Gene Expression and Phenotypes
Source: Cancers (Basel). 2023 Jan 21;15(3):674. doi: 10.3390/cancers15030674 (PMC9913820; doi:10.3390/cancers15030674)
Supplement: Supplementary file 1 [file cancers-15-00674-s001.zip › Supplementary Data S1 - Diet Composition.pdf]

Mod TestDiet® AIN-93G w/26% Broccoli Sprout Seeds/Red 5AJU

DESCRIPTION

Modification of TestDiet® AIN-93G with 26% broccoli sprout seeds and red dye.

Storage conditions are particularly critical to TestDiet® products, due to the absence of antioxidants or preservative agents. To provide maximum protection against possible changes during storage, store in a dry, cool location. Storage under refrigeration (2° C) is recommended. Maximum shelf life is six months. (If long term studies are involved, storing the diet at -20° C or colder may prolong shelf life.) Be certain to keep in air tight containers.

Product Forms Available*	***Catalog #***
1/2" Pellet	1814479

**Other Forms Available On Re*

INGREDIENTS (%)

Broccoli Sprout Seeds	26.0000
Casein - Vitamin Free	20.0000
Corn Starch	13.6986
Maltodextrin	13.2000
Sucrose	10.0000
Soybean Oil	7.0000
Powdered Cellulose	5.0000
AIN 93G Mineral Mix	3.5000
AIN 93 Vitamin Mix	1.0000
L-Cystine	0.3000
Choline Bitartrate	0.2500
Red Dye	0.0500
t-Butylhydroquinone	0.0014

FEEDING DIRECTIONS

Feed ad libitum. Plenty of fresh, clean water should be available at all times.

CAUTION:
Perishable - store properly upon receipt.
For laboratory animal use only; NOT for human consumption.

9/8/2010

NUTRITIONAL PROFILE [1]

Protein, %	***18.0***
Arginine, %	0.70
Histidine, %	0.52
Isoleucine, %	0.96
Leucine, %	1.73
Lysine, %	1.45
Methionine, %	0.52
Cystine, %	0.37
Phenylalanine, %	0.96
Tyrosine, %	1.01
Threonine, %	0.77
Tryptophan, %	0.22
Valine, %	1.14
Alanine, %	0.55
Aspartic Acid, %	1.29
Glutamic Acid, %	4.08
Glycine, %	0.39
Proline, %	2.36
Serine, %	1.10
Taurine, %	0.00
Fat, %	***7.0***
Cholesterol, ppm	0
Linoleic Acid, %	3.58
Linolenic Acid, %	0.55
Arachidonic Acid, %	0.00
Omega-3 Fatty Acids, %	0.55
Total Saturated Fatty A	1.05
Total Monounsaturated Fatty Acids, %	1.54
Polyunsaturated Fatty Acids, %	3.78
Fiber (max), %	***5.0***
Carbohydrates, %	***37.1***
Energy (kcal/g) [2]	***2.84***

From:	kcal	%
Protein	0.721	25.4
Fat (ether extract)	0.632	22.3
Carbohydrates	1.486	52.3

Minerals	
Calcium, %	0.51
Phosphorus, %	0.32
Phosphorus (available), %	0.16
Potassium, %	0.36
Magnesium, %	0.05
Sodium, %	0.12
Chloride, %	0.21
Fluorine, ppm	1.0
Iron, ppm	38
Zinc, ppm	35
Manganese, ppm	11
Copper, ppm	6.0
Cobalt, ppm	0.0
Iodine, ppm	0.21
Chromium, ppm	1.0
Molybdenum, ppm	0.14
Selenium, ppm	0.24

Vitamins	
Vitamin A, IU/g	4.0
Vitamin D-3 (added), IU/g	1.0
Vitamin E, IU/kg	81.6
Vitamin K (as menadione), ppm	0.75
Thiamin Hydrochloride, ppm	6.1
Riboflavin, ppm	6.7
Niacin, ppm	30
Pantothenic Acid, ppm	16
Folic Acid, ppm	2.1
Pyridoxine, ppm	5.8
Biotin, ppm	0.2
Vitamin B-12, mcg/kg	29
Choline Chloride, ppm	1,250
Ascorbic Acid, ppm	0.0

1. Formulation based on calculated values from the latest ingredient analysis information. Since nutrient composition of natural ingredients varies and some nutrient loss will occur due to manufacturing processes, analysis will differ accordingly. Nutrients expressed as percent of ration on an As-Fed basis except where otherwise indicated.
2. Energy (kcal/gm) - Sum of decimal fractions of protein, fat and carbohydrate x 4,9,4 kcal/gm respectively.

ISO 9001:2000 CERTIFIED

TestDiet
www.testdiet.com

Figure S1 Detailed dietary ingredients and nutritional profile of the customized broccoli sprout diet.
