# Supplementary material for: Peripubertal Nutritional Prevention of Cancer-Associated Gene Expression and Phenotypes
Source: Cancers (Basel). 2023 Jan 21;15(3):674. doi: 10.3390/cancers15030674 (PMC9913820; doi:10.3390/cancers15030674)
Supplement: Supplementary file 1 [file cancers-15-00674-s001.zip › cancers-2133837-supplementary.pdf]

cancers

MDPI

Supplementary Materials

Peripubertal Nutritional Prevention of Cancer-Associated Gene Expression and Phenotypes

Andrew Brane, Itika Arora and Trygve O. Tollefsbol

Table S1. Primer sequences for RT-qPCR.

GAPDH	GTGGAGTCATACTGGAACATGTAG<br>AATGGTGAAGGTCGGTGTG
P53	TGAAAATGTCTCCTGGCTCAG<br>CTAGCATTCAGGCCCTCATC
P21	GAAGAGACAACGGCACACT<br>CAGATCCACAGCGATATCCAG
BRCA2	TGTGTCATCCCTCTCCAGTATC<br>GATGCCTAAACCCAGAAAGAGT
Erich4	CCCCATCTCCAGTCCCA<br>TCAGGCCCTTATGTCTTCAG
Lman1I	ATCAAAGTAGATCCCGATGCC<br>GAGATGCAGATGAGAGTGACTG
Clec4e	TGAGAGCTGCGATATGTTACG<br>ATCCCACCACACAGAGAGA
Parp6	GCATACTTCATAATCTGGACAAGG<br>GTAGAGGTGTTTGGCTATCCC
Chrdl2	TCTCAGTTGTCCTGTCTTTGC<br>AGTGTGTCCTGTGTAGCTGTA
Pcsk1	TCACAGTTATCTCCCTGACGA<br>CCCAGAAGGCATTTGAATATGG
Slc51b	GCTGCTTCTTTCGATTTCTGTT<br>GCTTTGGTATTTTCGTGCAGA

Table S2. Antibodies for Western blots (File S1).

P53	Anti-p53 antibody (ab131442)
P21	Recombinant Anti-p21 antibody [EPR18021] (ab188224)
BRCA2	Anti-BRCA2 antibody (ab216972)
ActB	β-Actin (13E5) Rabbit mAb #4970

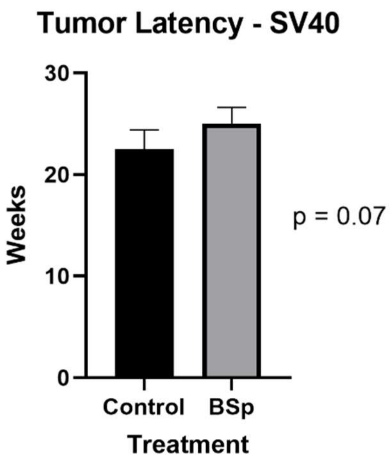

Figure S1. Average tumor latency observed in SV40 mice. BSp-treated mice developed tumors approximately 2 weeks later than controls in this mouse model. $p = 0.07$ and $n = 24$.

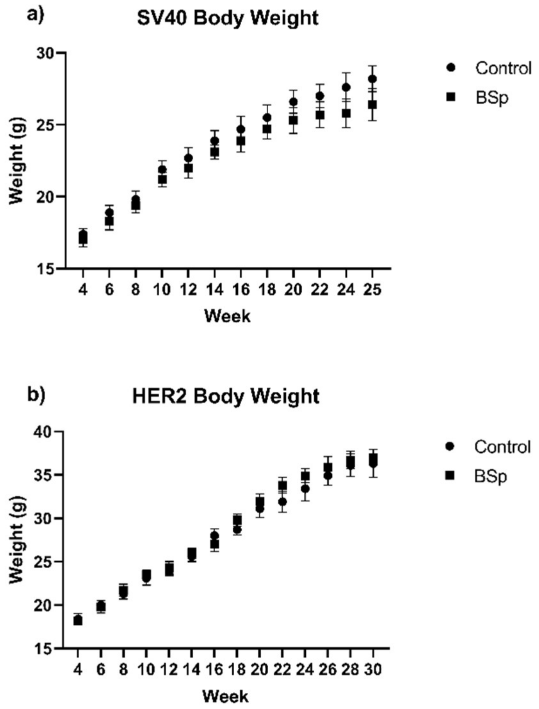

Figure S2. Mouse body weight for SV40 (**a**) and HER2/neu (**b**) mice. Treatment did not have a significant effect on total body weight in either mouse strain. n = 24 for both strains.

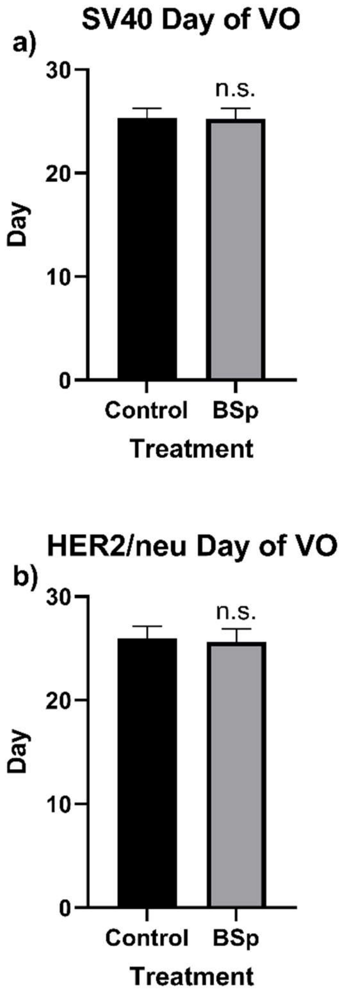

Figure S3. Average day of vaginal opening (VO) for SV40 (**a**) and HER2/neu (**b**) mice. Treatment did not have a significant effect on VO in either mouse strain. n = 24 for both strains. n.s., not significant.

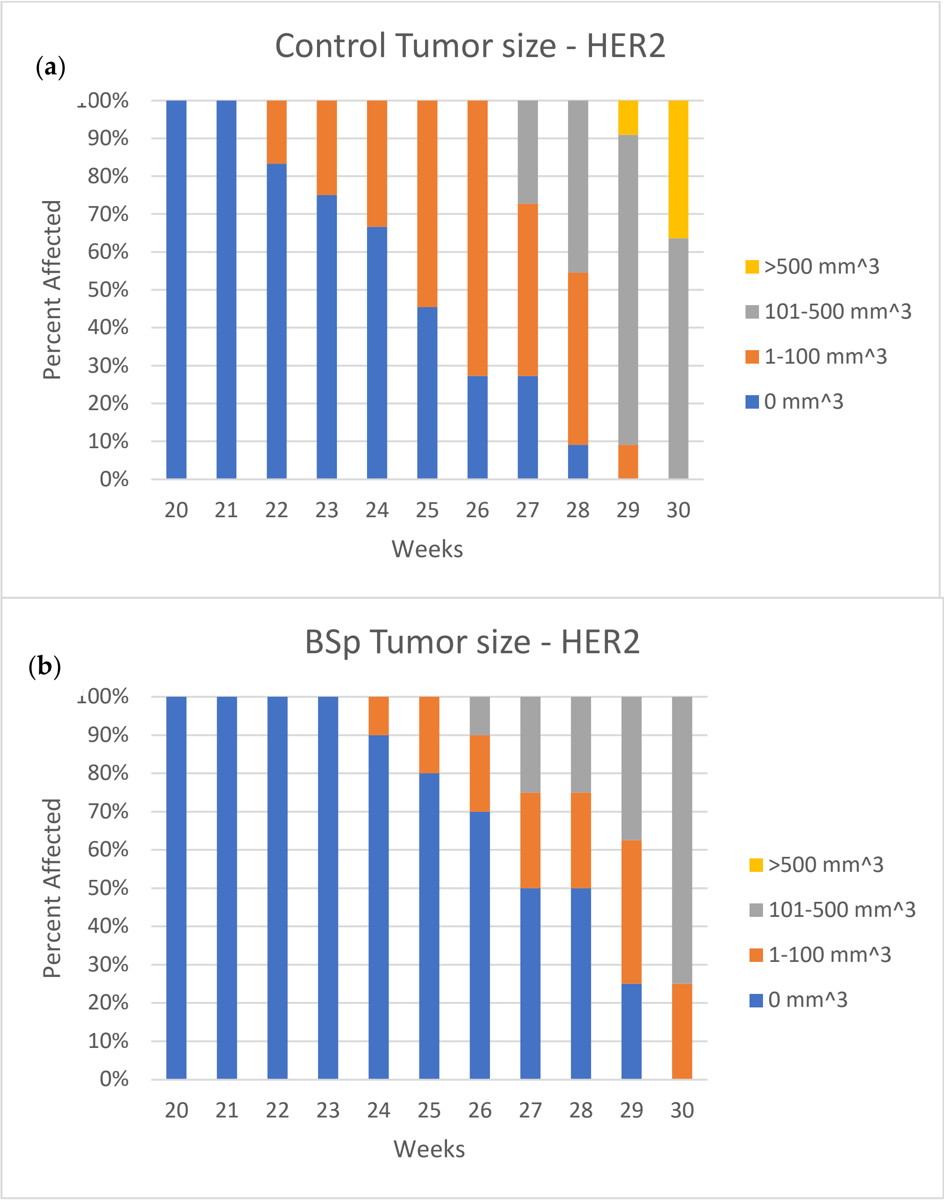

Figure S4. Approximate tumor size for HER2/neu mice in both control (**a**) and BSp (**b**) fed mice. n = 24 for both groups.

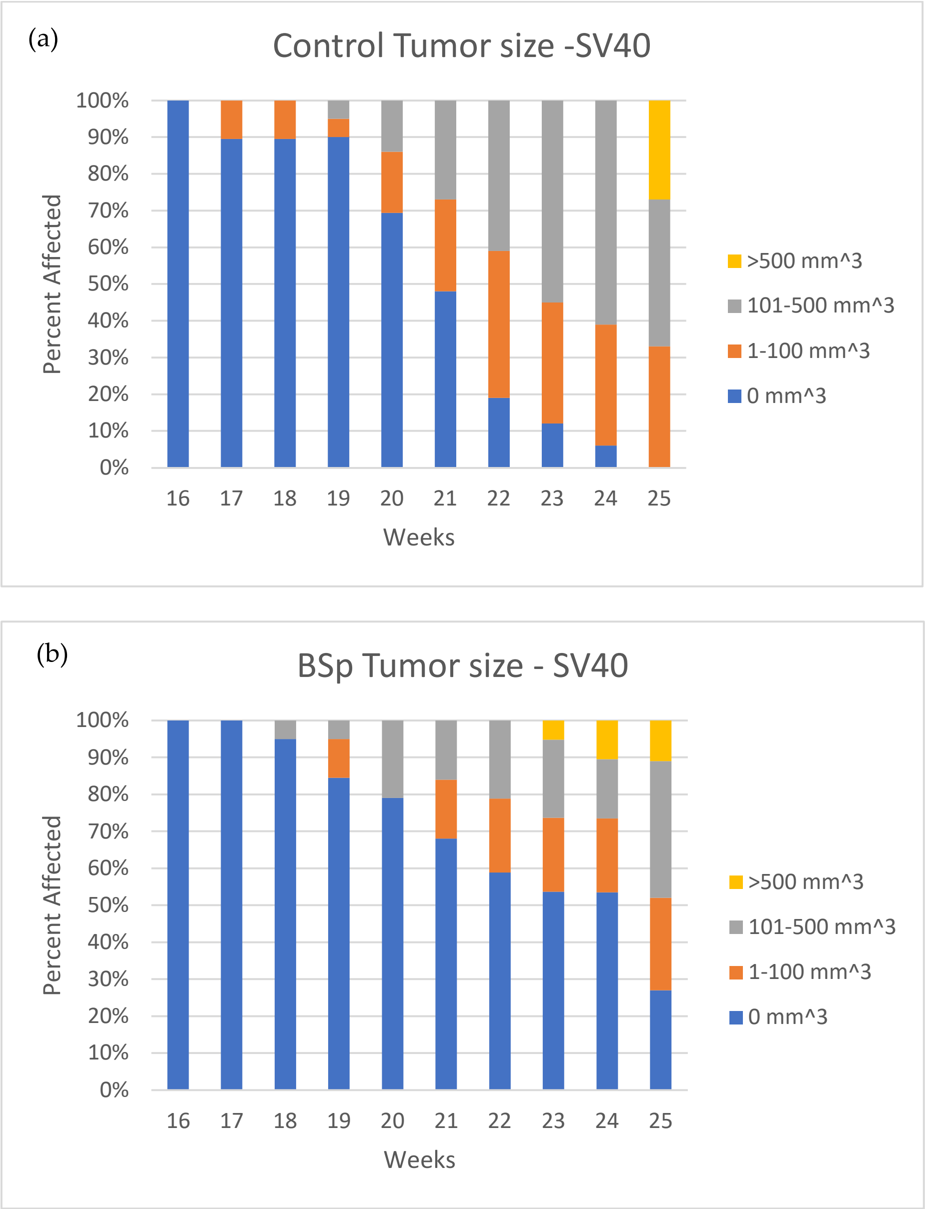

Figure S5. Approximate tumor size for SV40 mice in both control (**a**) and BSp (**b**) fed mice. $n = 24$ for both groups.

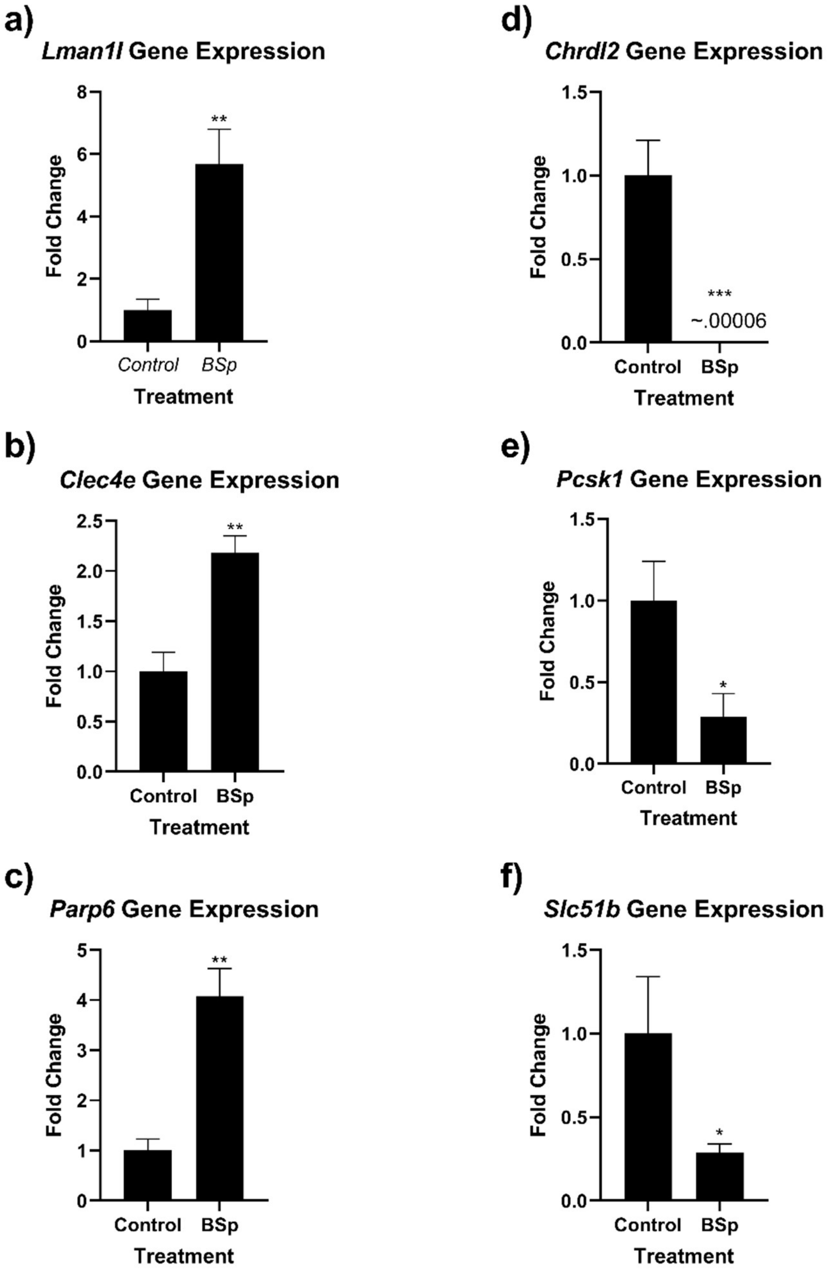

Figure S6. PCR verification of tumor suppressors *Lman1l* (**a**), *Clec4e* (**b**), *Parp6* (**c**), as well as oncogenes *Chrdl2* (**d**), *Pcsk1* (**e**), and *Slc51b* (**f**). $n = 10$ for all RT-qPCR. * = $p < 0.05$, ** = $p < 0.01$, *** = $p < 0.001$.

a)

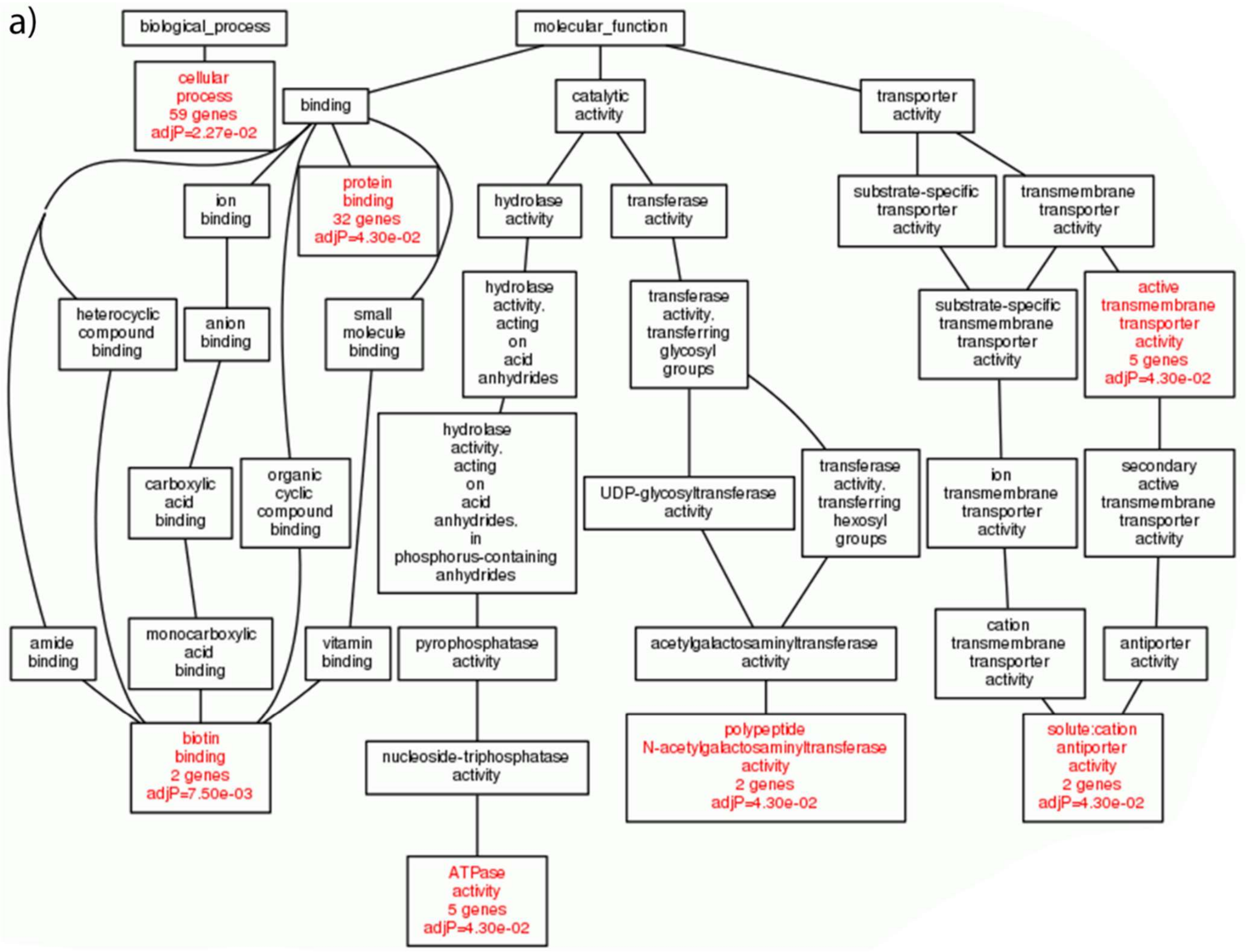
biological_process
molecular_function
cellular process 59 genes adjP=2.27e-02
binding
catalytic activity
transporter activity
ion binding
protein binding 32 genes adjP=4.30e-02
hydrolase activity
transferase activity
substrate-specific transporter activity
transmembrane transporter activity
heterocyclic compound binding
anion binding
small molecule binding
hydrolase activity, acting on acid anhydrides
transferase activity, transferring glycosyl groups
substrate-specific transmembrane transporter activity
active transmembrane transporter activity 5 genes adjP=4.30e-02
hydrolase activity, acting on acid anhydrides, in phosphorus-containing anhydrides
carboxylic acid binding
organic cyclic compound binding
UDP-glycosyltransferase activity
transferase activity, transferring hexosyl groups
ion transmembrane transporter activity
secondary active transmembrane transporter activity
amide binding
monocarboxylic acid binding
vitamin binding
pyrophosphatase activity
acetylgalactosaminyltransferase activity
cation transmembrane transporter activity
antiporter activity
biotin binding 2 genes adjP=7.50e-03
nucleoside-triphosphatase activity
polypeptide N-acetylgalactosaminyltransferase activity 2 genes adjP=4.30e-02
solute:cation antiporter activity 2 genes adjP=4.30e-02
ATPase activity 5 genes adjP=4.30e-02

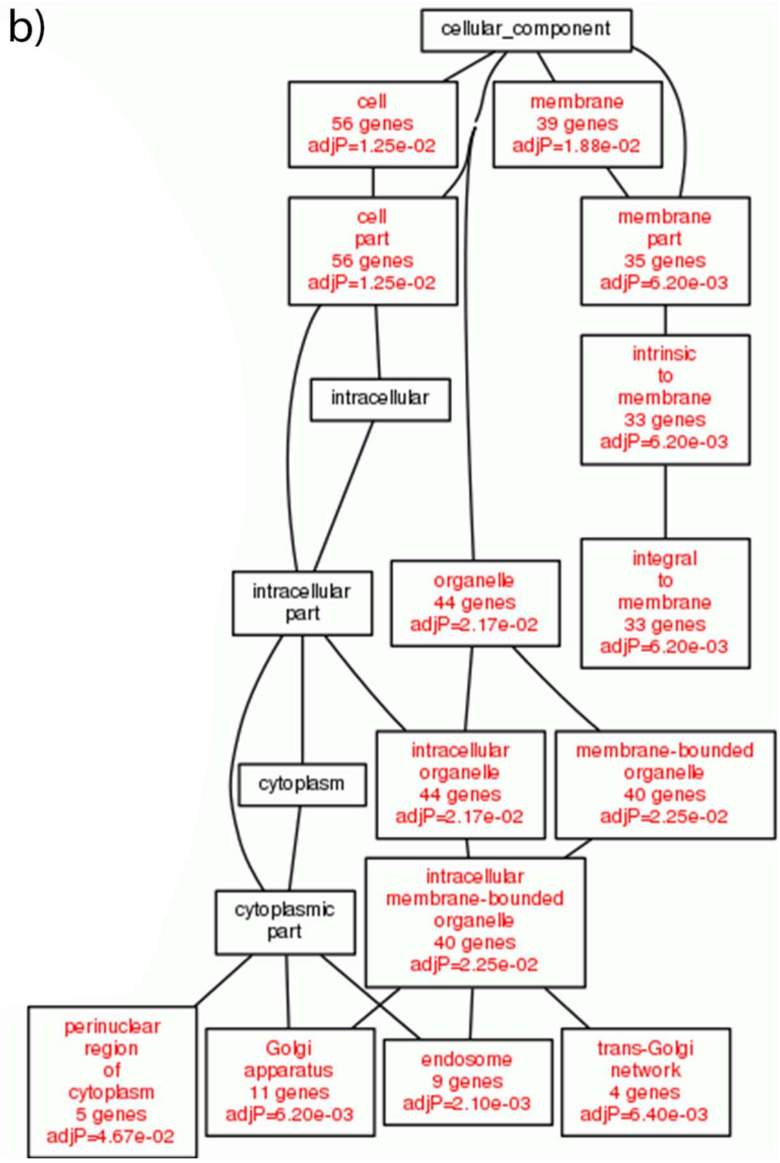

Figure S7. Gene ontology analysis for downregulated genes identified by RNA sequencing. Overall, there were 21 significantly upregulated groups of genes within the overarching groups of biological processes (**a**), molecular function (**a**), and cellular components (**b**). Significant groupings of genes are identified in red. P-value cutoff for gene ontology analysis was $p < 0.05$.

a)

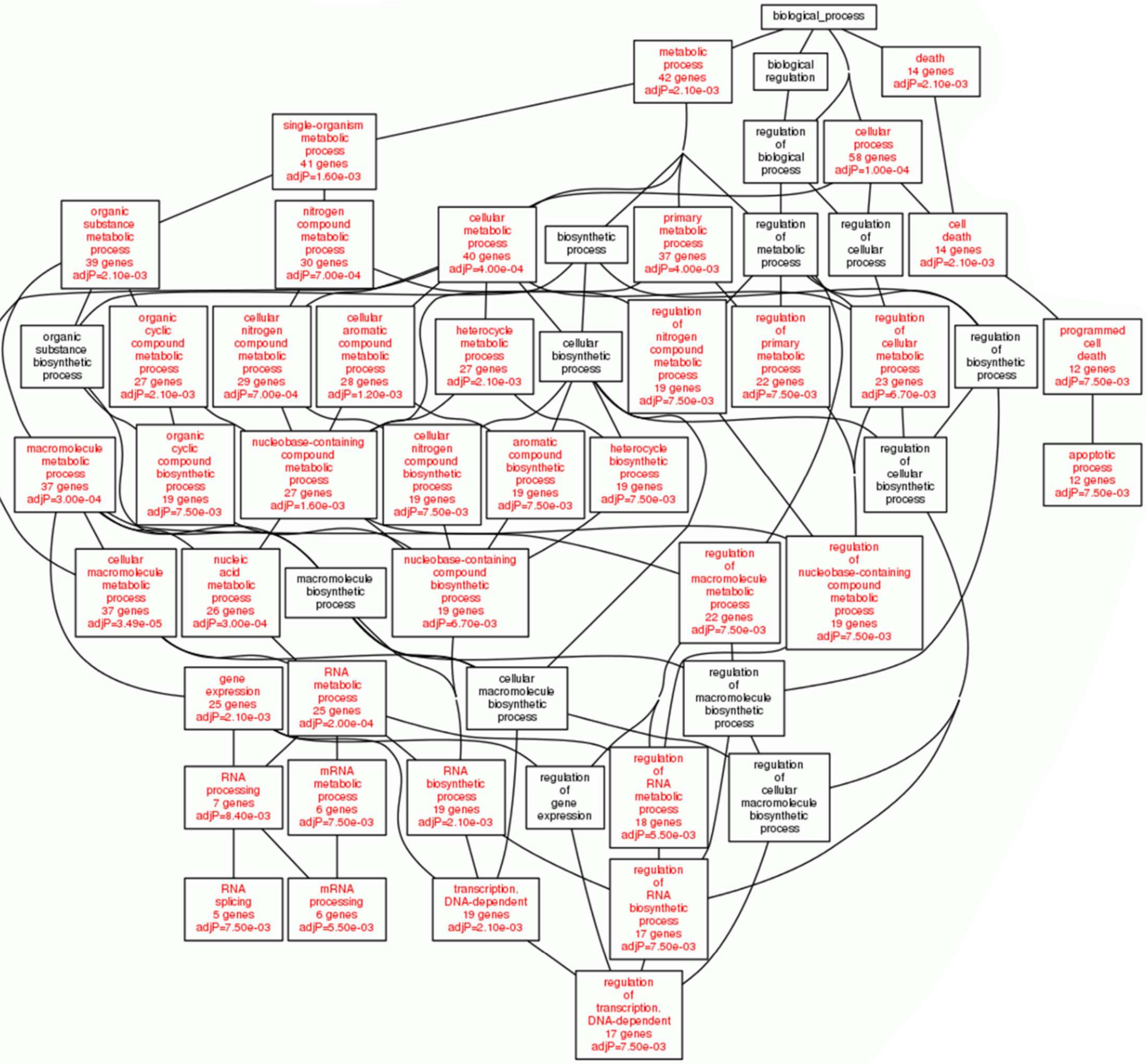
biological_process
metabolic process 42 genes adjP=2.10e-03
biological regulation
death 14 genes adjP=2.10e-03
single-organism metabolic process 41 genes adjP=1.60e-03
regulation of biological process
cellular process 58 genes adjP=1.00e-04
organic substance metabolic process 39 genes adjP=2.10e-03
nitrogen compound metabolic process 30 genes adjP=7.00e-04
cellular metabolic process 40 genes adjP=4.00e-04
biosynthetic process
primary metabolic process 37 genes adjP=4.00e-03
regulation of metabolic process
regulation of cellular process
cell death 14 genes adjP=2.10e-03
organic substance biosynthetic process
organic cyclic compound metabolic process 27 genes adjP=2.10e-03
cellular nitrogen compound metabolic process 29 genes adjP=7.00e-04
cellular aromatic compound metabolic process 28 genes adjP=1.20e-03
heterocycle metabolic process 27 genes adjP=2.10e-03
cellular biosynthetic process
regulation of nitrogen compound metabolic process 19 genes adjP=7.50e-03
regulation of primary metabolic process 22 genes adjP=7.50e-03
regulation of cellular metabolic process 23 genes adjP=6.70e-03
regulation of biosynthetic process
programmed cell death 12 genes adjP=7.50e-03
macromolecule metabolic process 37 genes adjP=3.00e-04
organic cyclic compound biosynthetic process 19 genes adjP=7.50e-03
nucleobase-containing compound metabolic process 27 genes adjP=1.60e-03
cellular nitrogen compound biosynthetic process 19 genes adjP=7.50e-03
aromatic compound biosynthetic process 19 genes adjP=7.50e-03
heterocycle biosynthetic process 19 genes adjP=7.50e-03
regulation of cellular biosynthetic process
apoptotic process 12 genes adjP=7.50e-03
cellular macromolecule metabolic process 37 genes adjP=3.49e-05
nucleic acid metabolic process 26 genes adjP=3.00e-04
macromolecule biosynthetic process
nucleobase-containing compound biosynthetic process 19 genes adjP=6.70e-03
regulation of macromolecule metabolic process 22 genes adjP=7.50e-03
regulation of nucleobase-containing compound metabolic process 19 genes adjP=7.50e-03
gene expression 25 genes adjP=2.10e-03
RNA metabolic process 25 genes adjP=2.00e-04
cellular macromolecule biosynthetic process
regulation of macromolecule biosynthetic process
RNA processing 7 genes adjP=8.40e-03
mRNA metabolic process 6 genes adjP=7.50e-03
RNA biosynthetic process 19 genes adjP=2.10e-03
regulation of gene expression
regulation of RNA metabolic process 18 genes adjP=5.50e-03
regulation of cellular macromolecule biosynthetic process
RNA splicing 5 genes adjP=7.50e-03
mRNA processing 6 genes adjP=5.50e-03
transcription, DNA-dependent 19 genes adjP=2.10e-03
regulation of RNA biosynthetic process 17 genes adjP=7.50e-03
regulation of transcription, DNA-dependent 17 genes adjP=7.50e-03

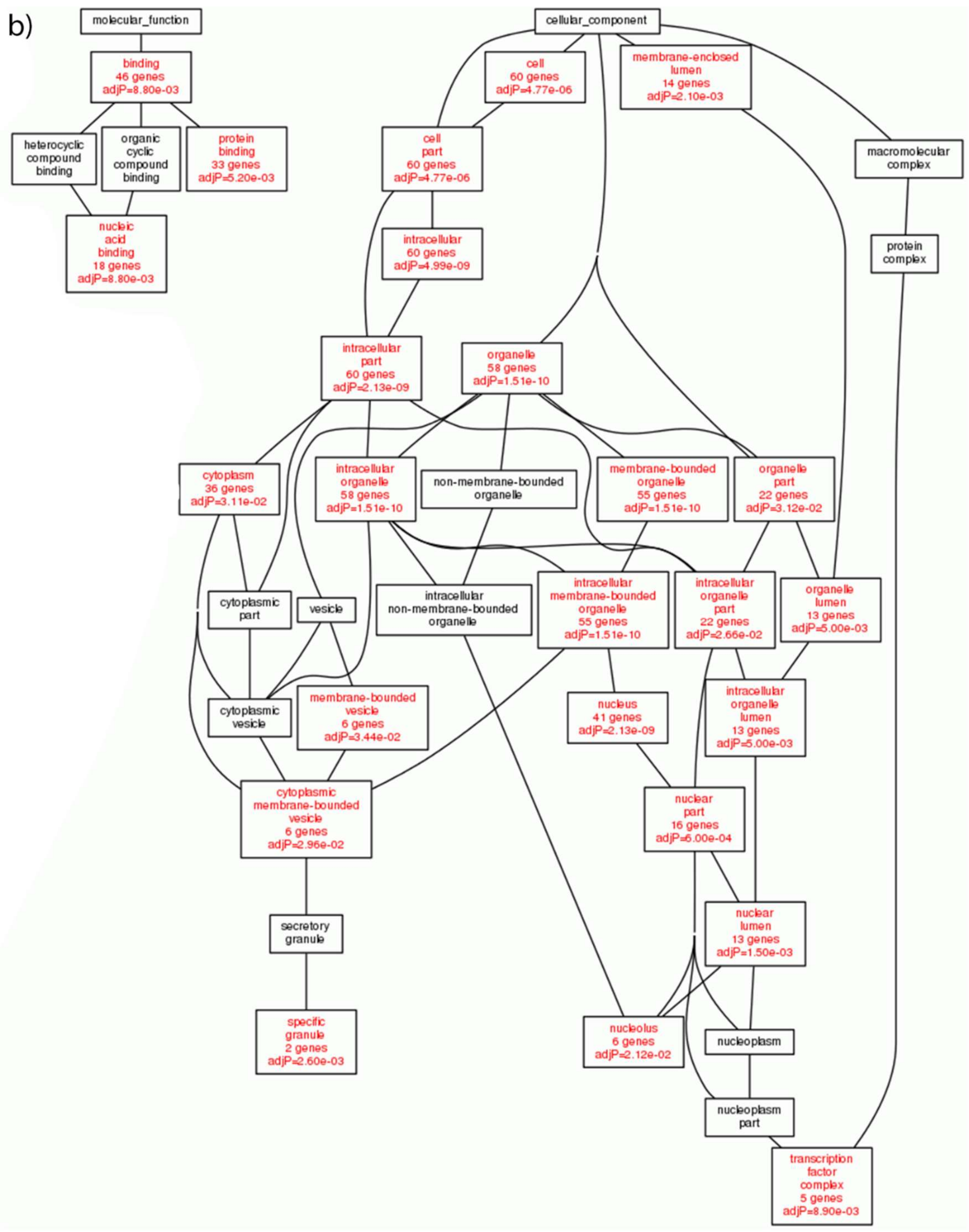

Figure S8. Gene ontology analysis for upregulated genes identified by RNA sequencing. Overall, there were 65 significantly upregulated groups of genes within the overarching groups of biological processes (**a**), molecular function (**b**), and cellular components (**b**). Significant groupings of genes are identified in red. P-value cutoff for gene ontology analysis was $p < 0.05$.
