# Supplementary figures and images for: Peripubertal Nutritional Prevention of Cancer-Associated Gene Expression and Phenotypes
Source: Cancers (Basel). 2023 Jan 21;15(3):674. doi: 10.3390/cancers15030674 (PMC9913820; doi:10.3390/cancers15030674)

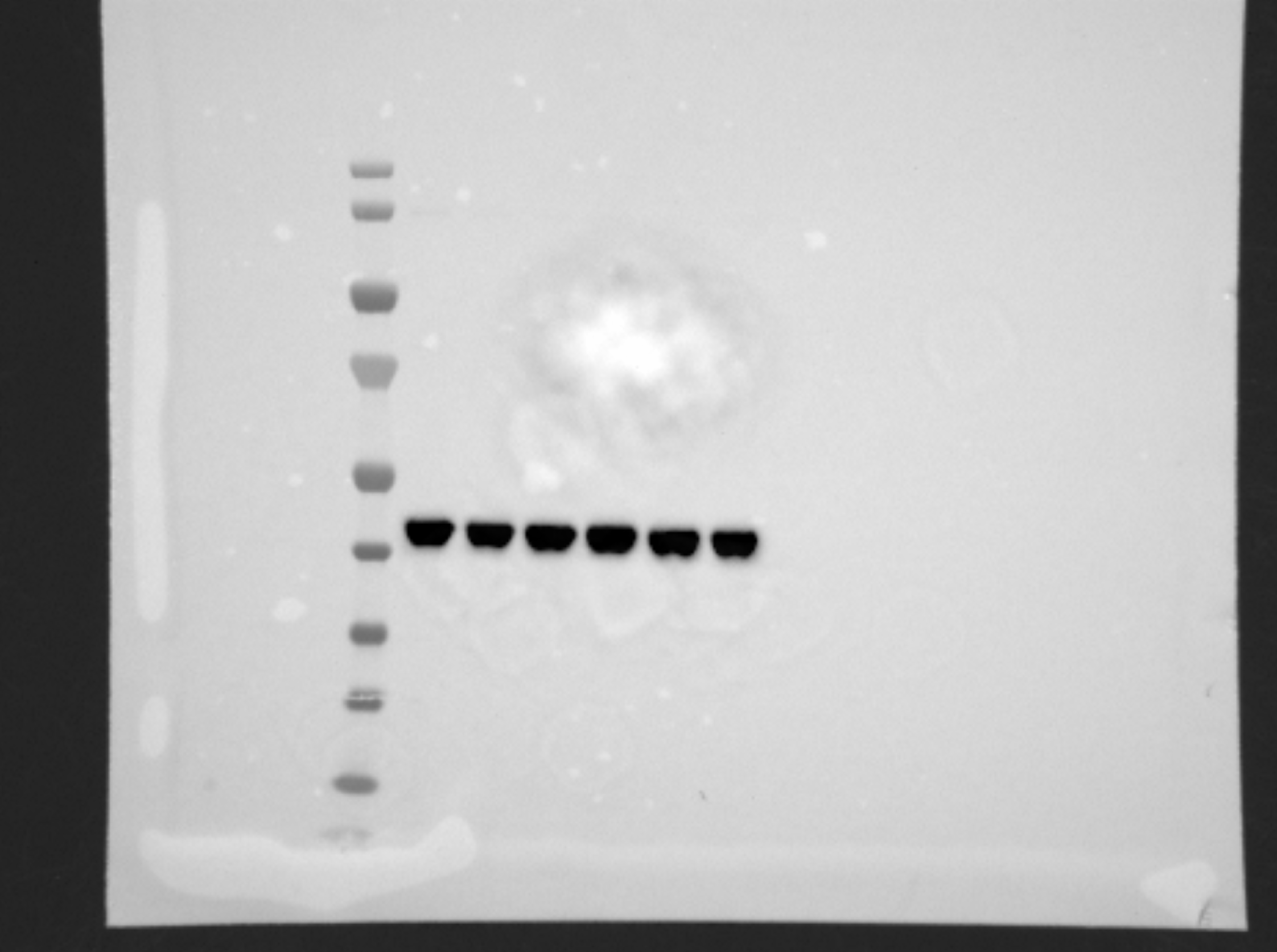

Supplement: Supplementary file 1 [file cancers-15-00674-s001.zip › File S1/brca2 - ActB samples 1-3.tif]

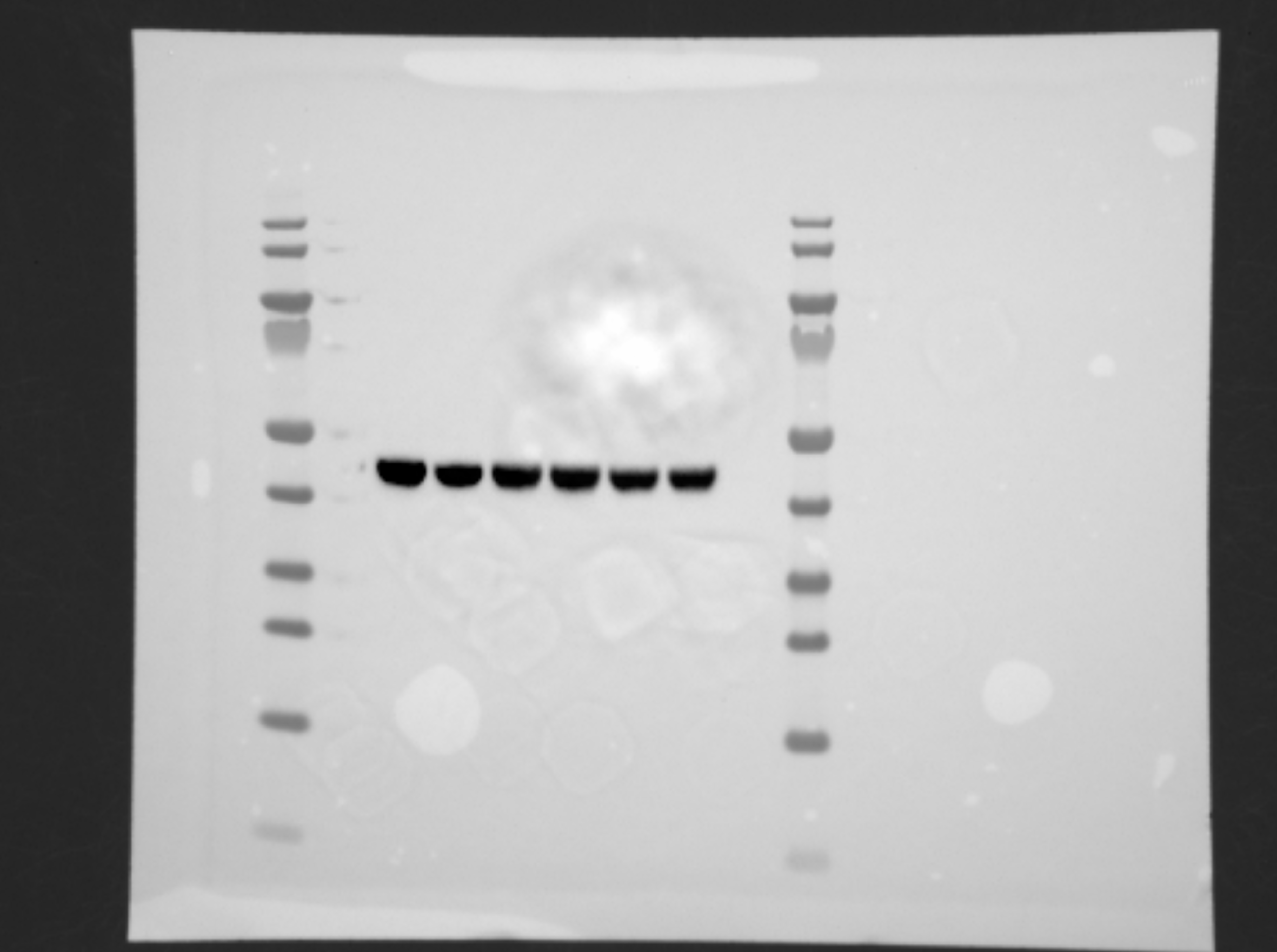

Supplement: Supplementary file 1 [file cancers-15-00674-s001.zip › File S1/brca2 - ActB samples 4-6.tif]

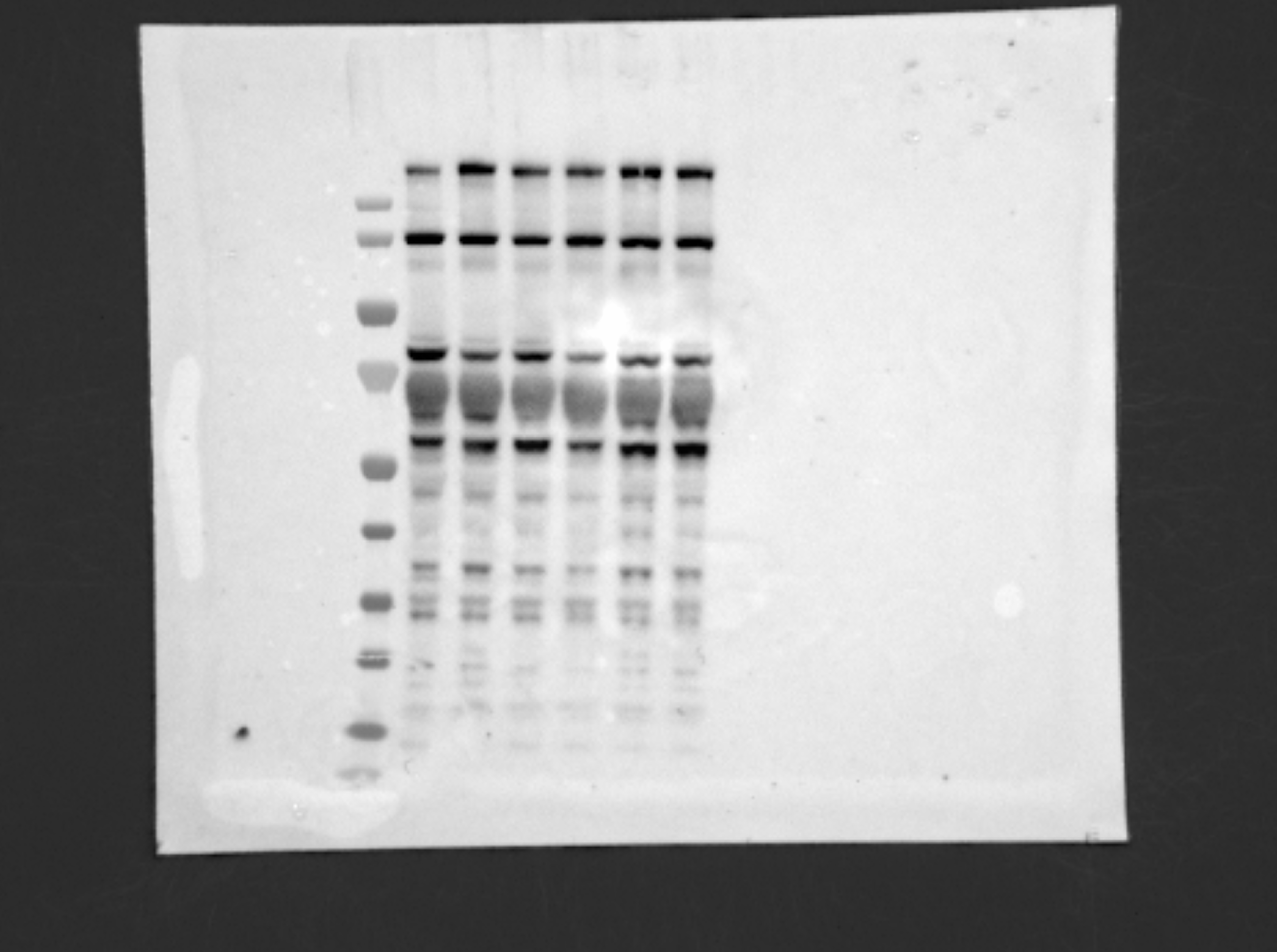

Supplement: Supplementary file 1 [file cancers-15-00674-s001.zip › File S1/brca2 samples 1-3.tif]

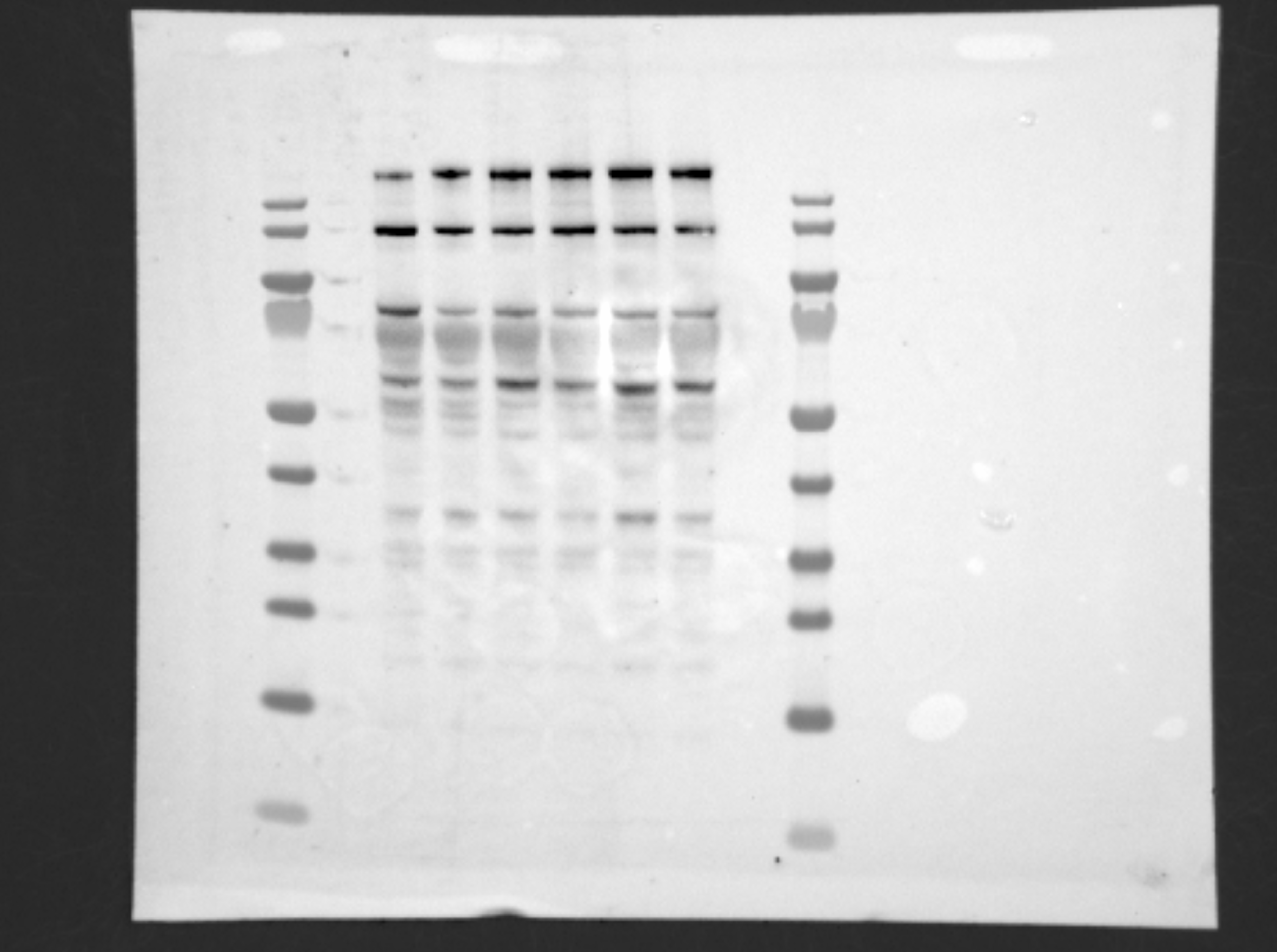

Supplement: Supplementary file 1 [file cancers-15-00674-s001.zip › File S1/brca2 samples 4-6.tif]

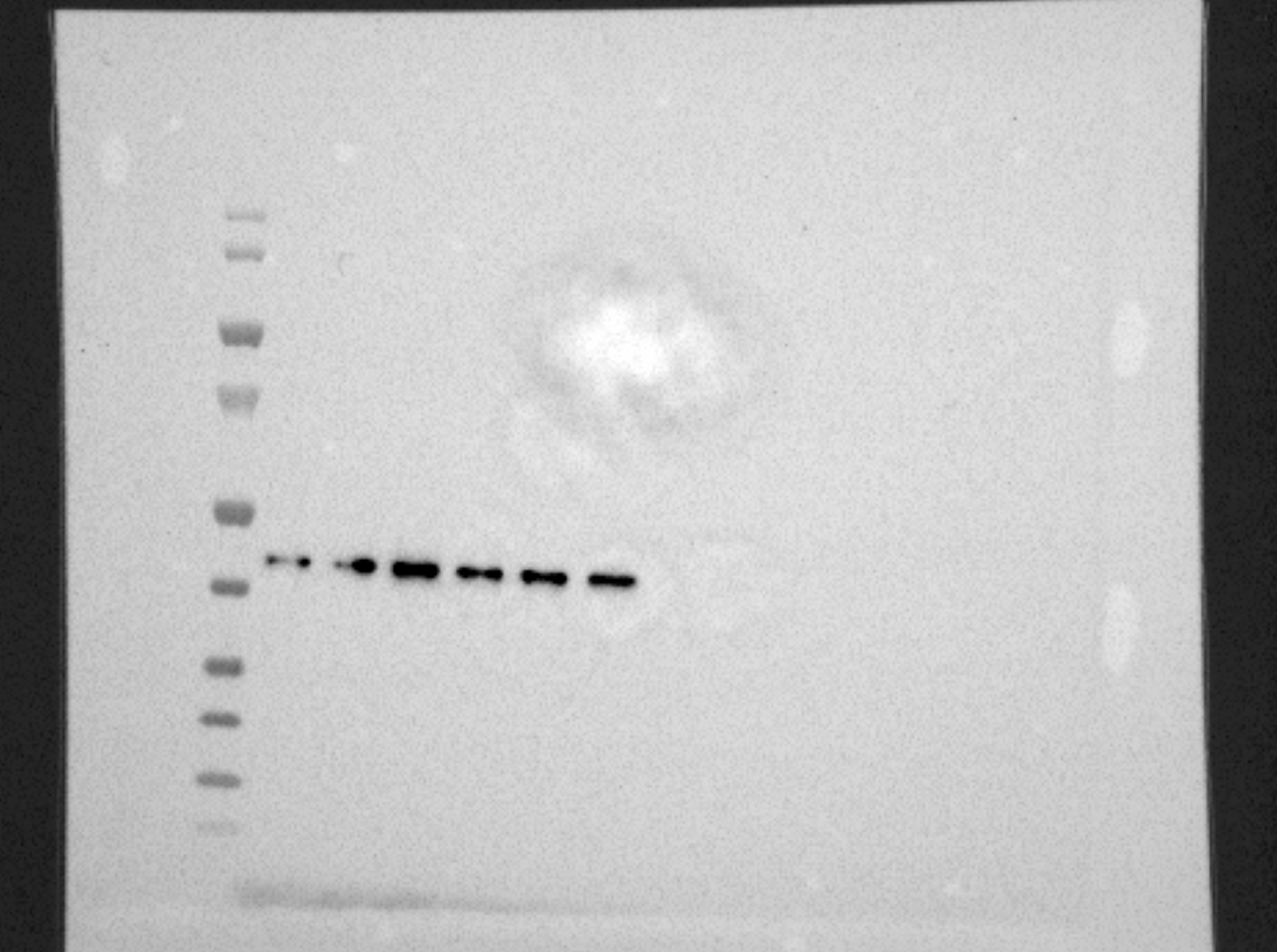

Supplement: Supplementary file 1 [file cancers-15-00674-s001.zip › File S1/p21 - ActB samples 1-3.tif]

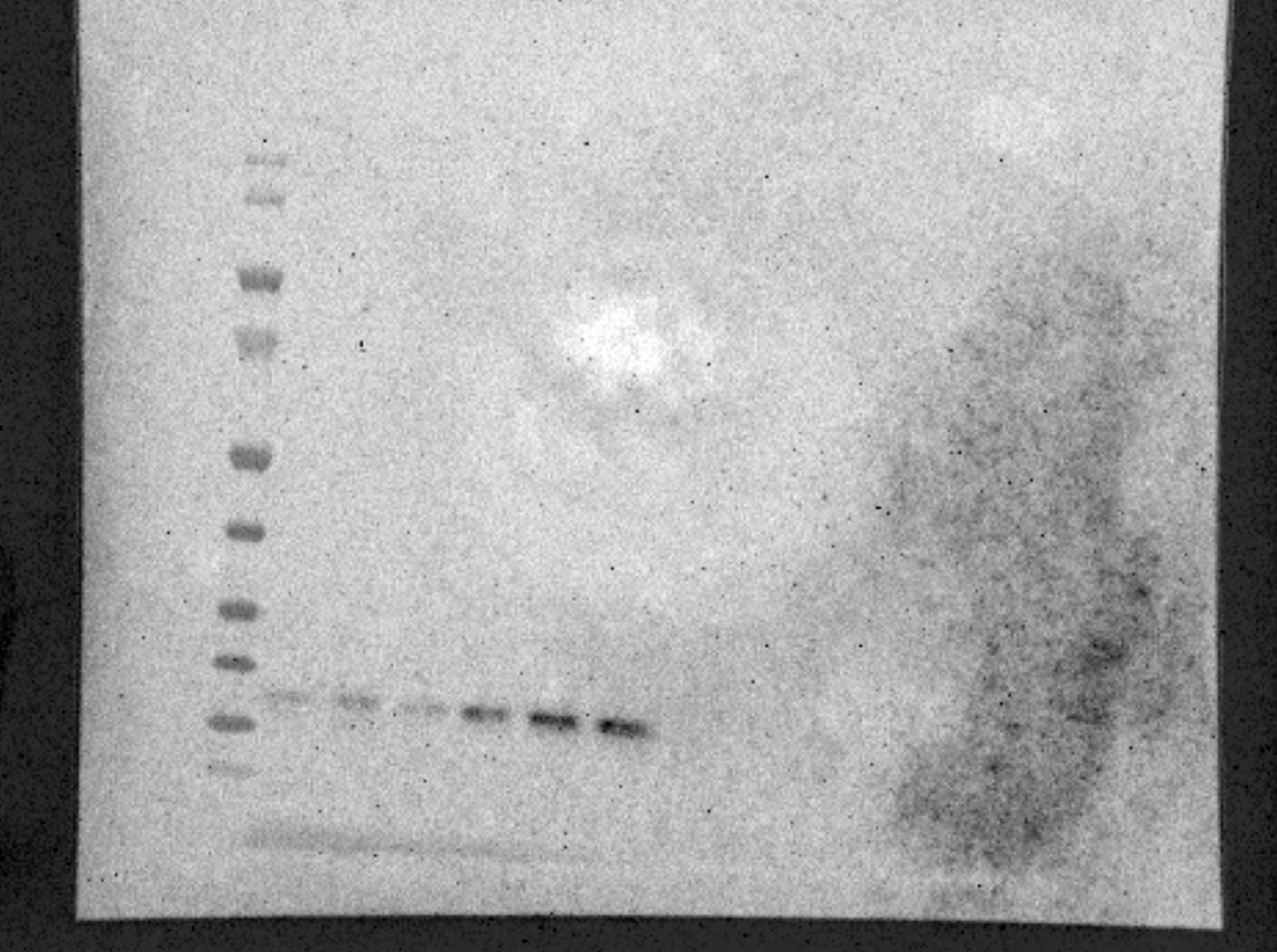

Supplement: Supplementary file 1 [file cancers-15-00674-s001.zip › File S1/p21 - samples 1-3.tif]

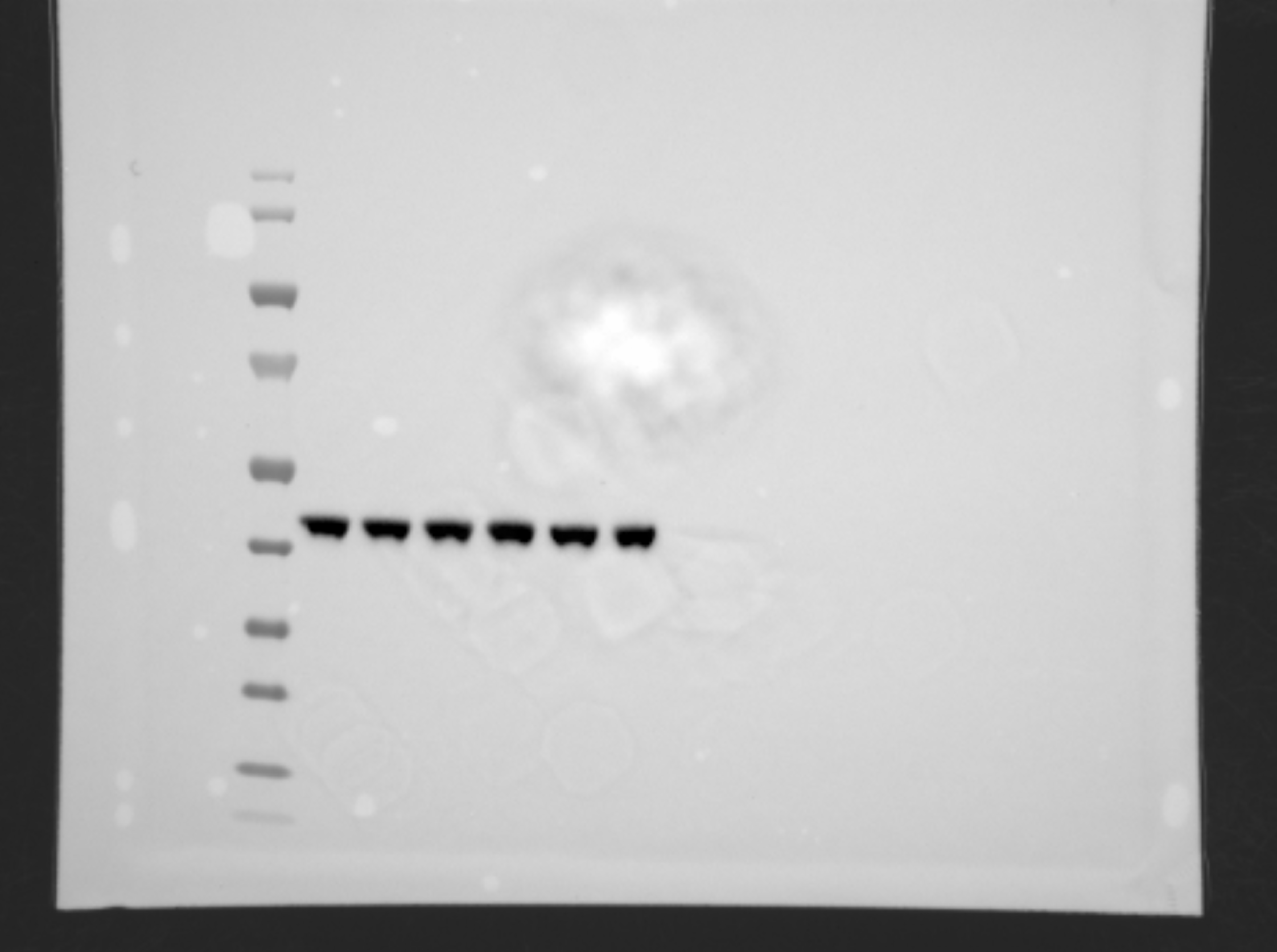

Supplement: Supplementary file 1 [file cancers-15-00674-s001.zip › File S1/p21 ActB samples 4-6.tif]

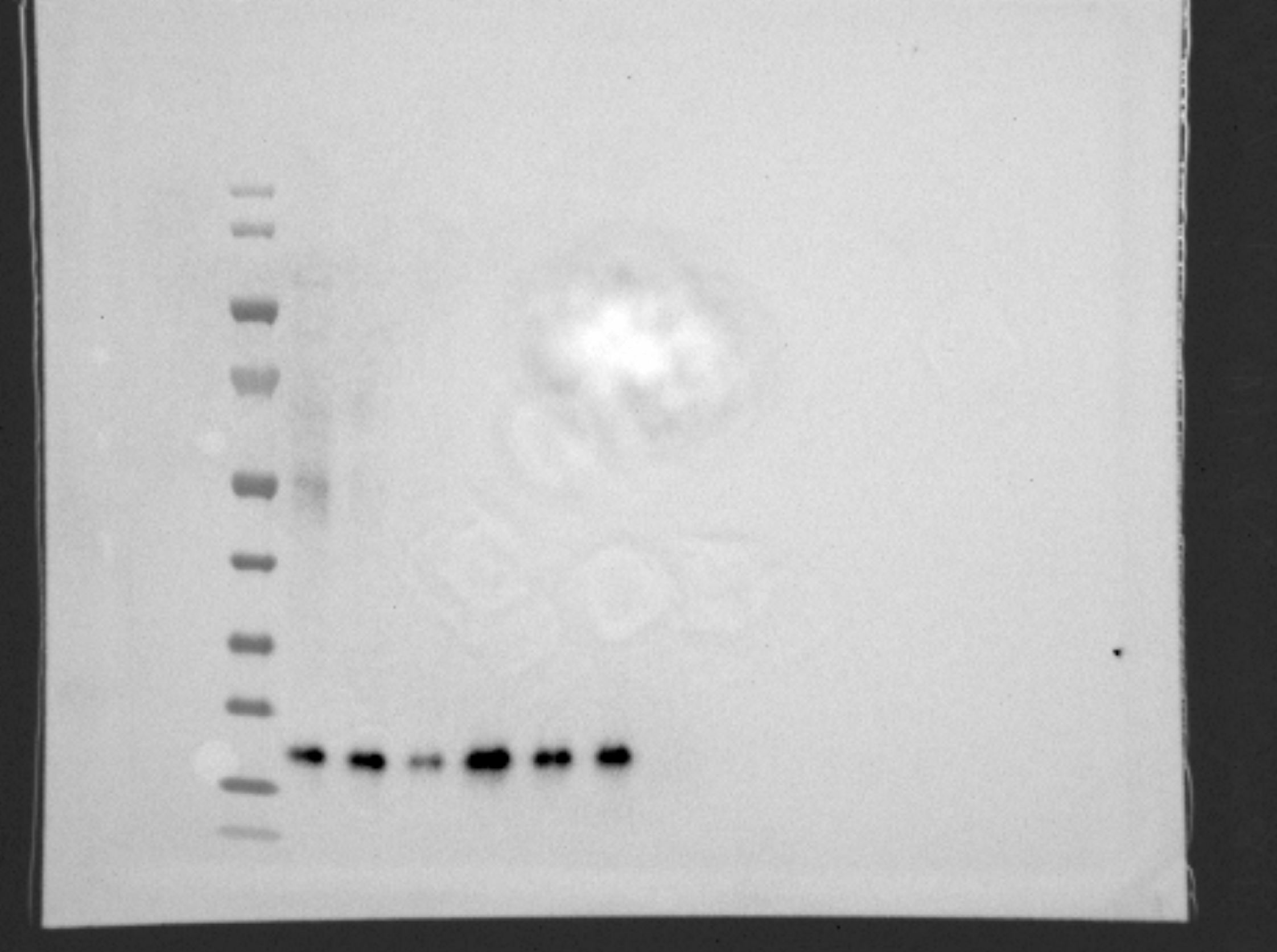

Supplement: Supplementary file 1 [file cancers-15-00674-s001.zip › File S1/p21 samples 4-6.tif]

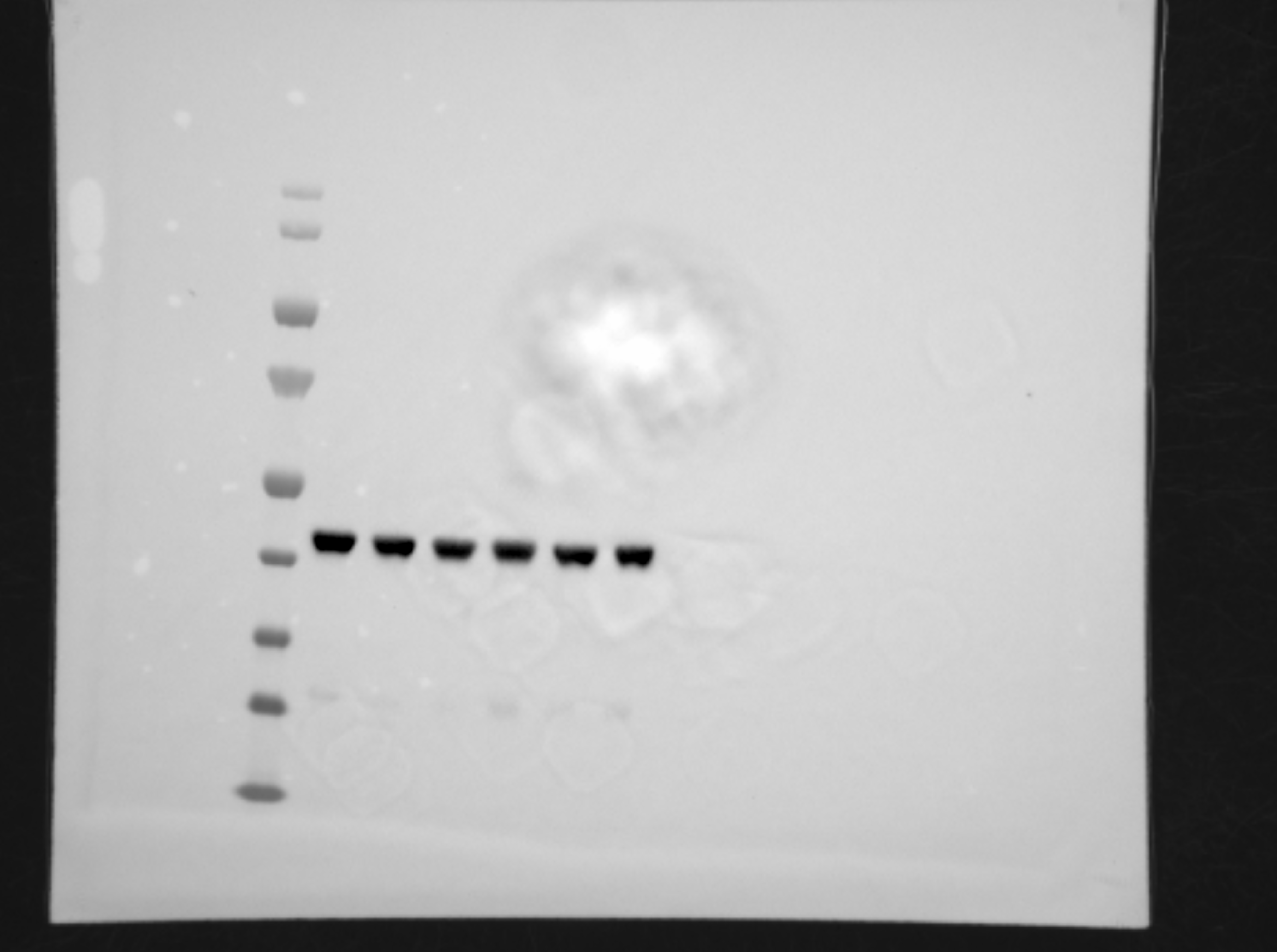

Supplement: Supplementary file 1 [file cancers-15-00674-s001.zip › File S1/p53 - ActB samples 1-3.tif]

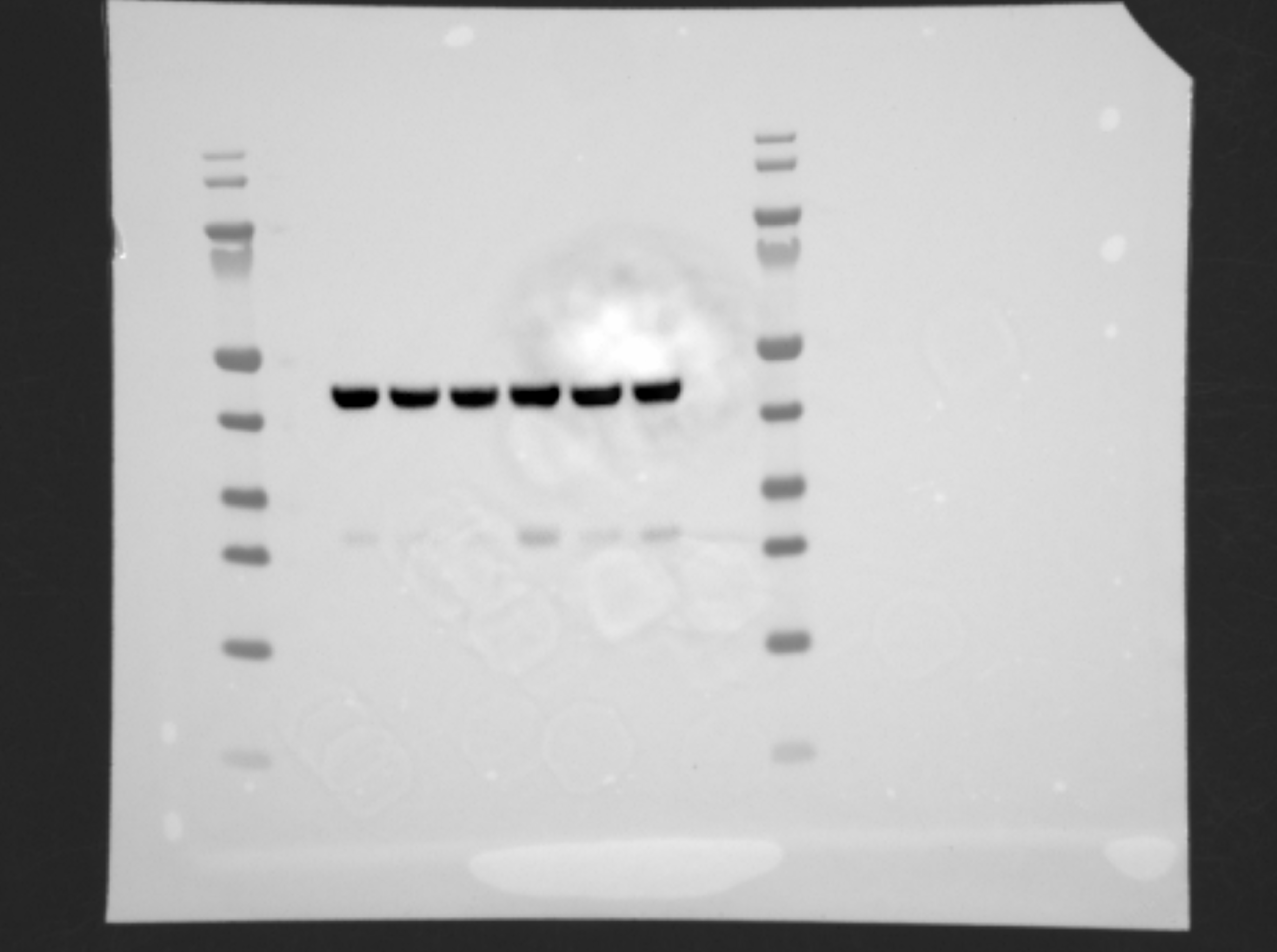

Supplement: Supplementary file 1 [file cancers-15-00674-s001.zip › File S1/p53 ActB samples 4-6.tif]

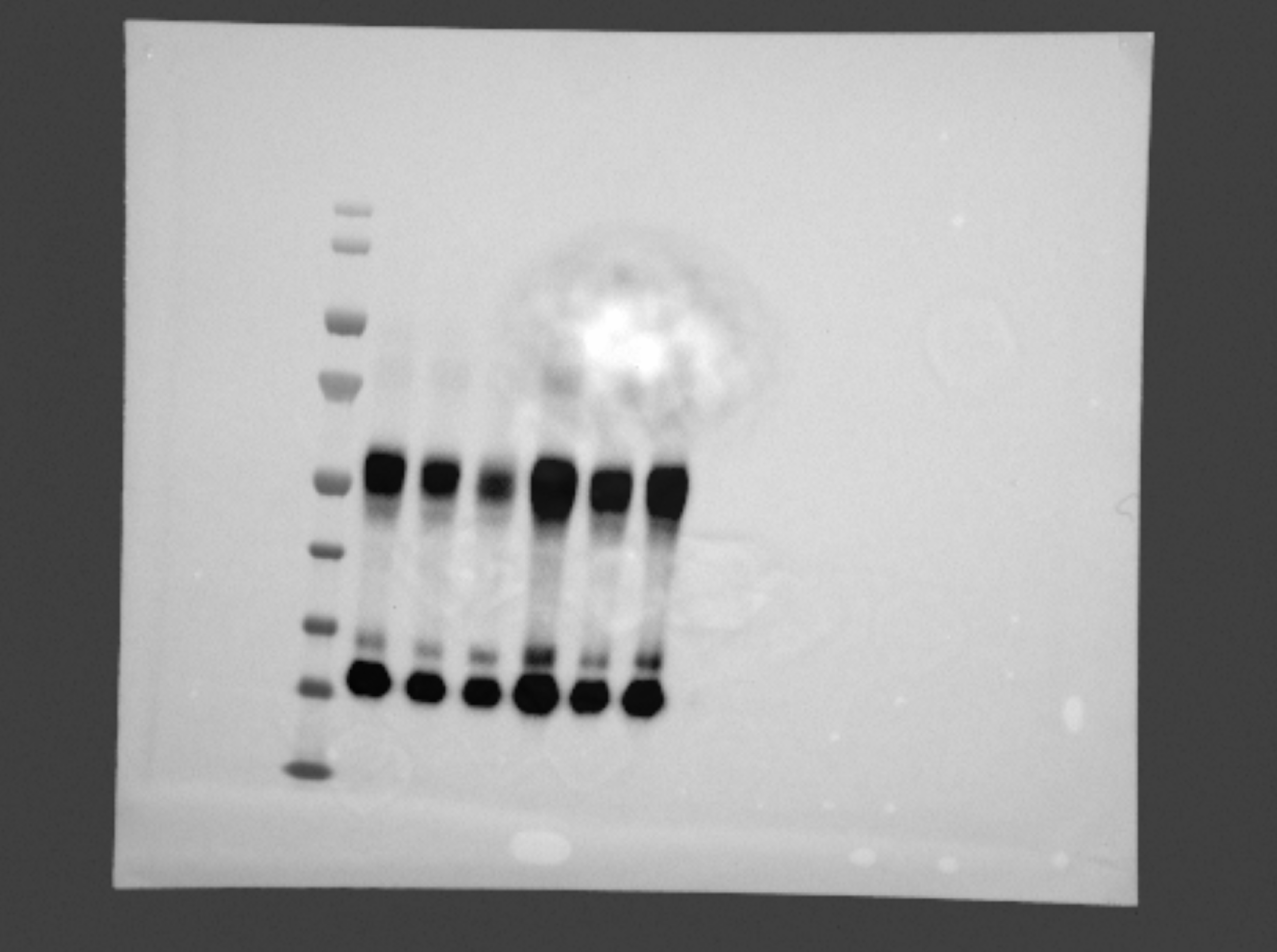

Supplement: Supplementary file 1 [file cancers-15-00674-s001.zip › File S1/p53 samples 1-3.tif]

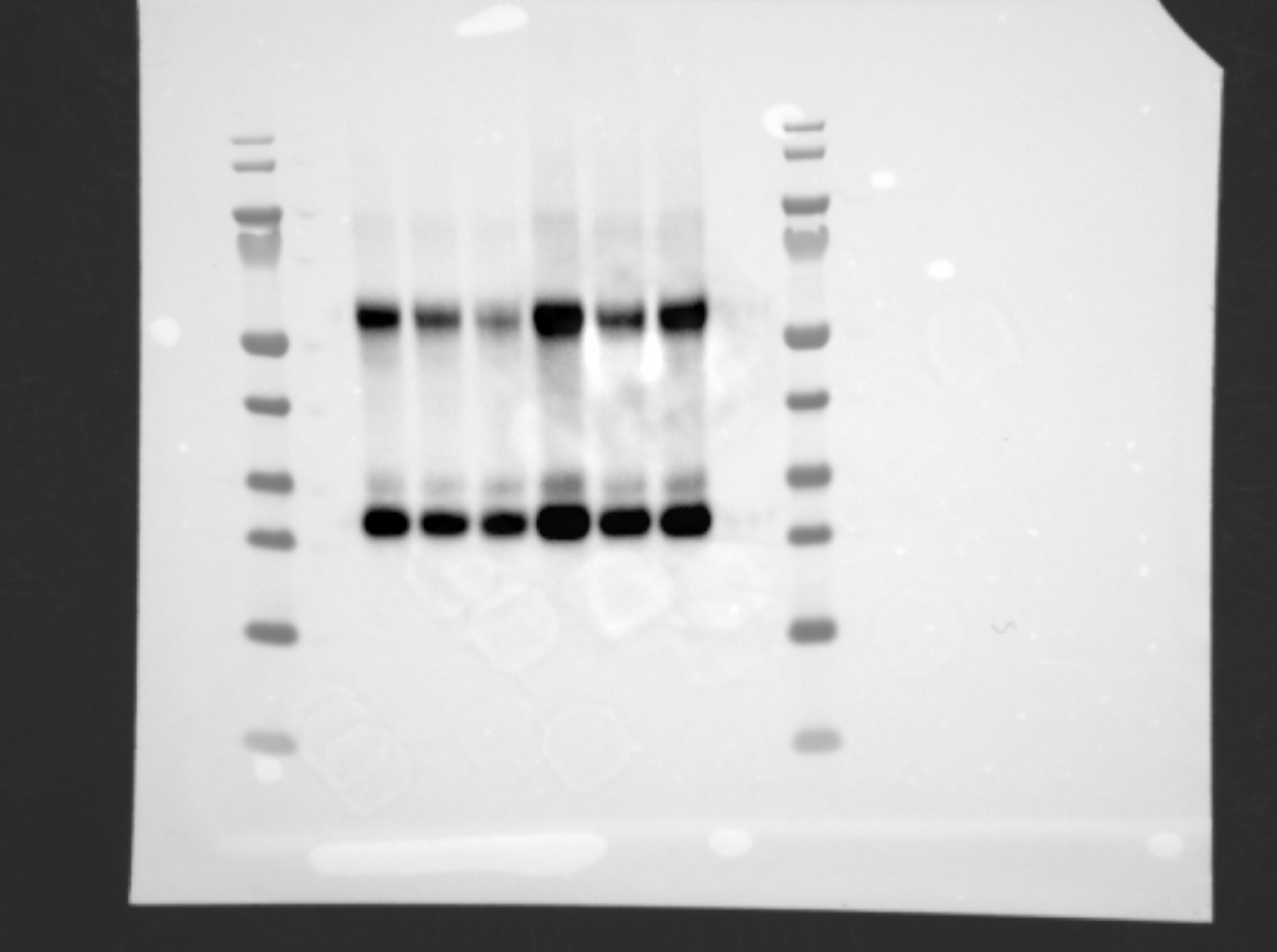

Supplement: Supplementary file 1 [file cancers-15-00674-s001.zip › File S1/p53 samples 4-6.tif]
